# Causal relationship between resting-state networks and depression: a bidirectional two-sample mendelian randomization study

**DOI:** 10.1186/s12888-024-05857-2

**Published:** 2024-05-29

**Authors:** Dongmiao Huang, Yuelin Wu, Jihui Yue, Xianglan Wang

**Affiliations:** Department of Psychiatry, the Fifth Affiliated Hospital of Sun Yat-sen University, No. 52, East Meihua Road, Zhuhai City, Guangdong Province 519000 China

**Keywords:** Bidirectional two-sample mendelian randomization, Depressive disorders, Resting-state brain network, Neuroimaging

## Abstract

**Background:**

Cerebral resting-state networks were suggested to be strongly associated with depressive disorders. However, the causal relationship between cerebral networks and depressive disorders remains controversial. In this study, we aimed to investigate the effect of resting-state networks on depressive disorders using a bidirectional Mendelian randomization (MR) design.

**Methods:**

Updated summary-level genome-wide association study (GWAS) data correlated with resting-state networks were obtained from a meta-analysis of European-descent GWAS from the Complex Trait Genetics Lab. Depression-related GWAS data were obtained from the FinnGen study involving participants with European ancestry. Resting-state functional magnetic resonance imaging and multiband diffusion imaging of the brain were performed to measure functional and structural connectivity in seven well-known networks. Inverse-variance weighting was used as the primary estimate, whereas the MR-Pleiotropy RESidual Sum and Outliers (PRESSO), MR-Egger, and weighted median were used to detect heterogeneity, sensitivity, and pleiotropy.

**Results:**

In total, 20,928 functional and 20,573 structural connectivity data as well as depression-related GWAS data from 48,847 patients and 225,483 controls were analyzed. Evidence for a causal effect of the structural limbic network on depressive disorders was found in the inverse variance–weighted limbic network (odds ratio, $28.21$; 95% confidence interval, $3.32-239.54$; $\text{P}=0.002$), whereas the causal effect of depressive disorders on SC LN was not found(OR=1.0025; CI,1.0005-1.0046; *P*=0.012). No significant associations between functional connectivity of the resting-state networks and depressive disorders were found in this MR study.

**Conclusions:**

These results suggest that genetically determined structural connectivity of the limbic network has a causal effect on depressive disorders and may play a critical role in its neuropathology.

**Supplementary Information:**

The online version contains supplementary material available at 10.1186/s12888-024-05857-2.

## Background

Depressive disorders include major depressive disorder(MDD) and dysthymia, which are the leading cause of disability and are characterized by significantly decreased mood, lack of interest, anhedonia, and reduced energy [1]. It causes functional impairment and mental distress and can even lead to suicidal ideation, attempts, and behaviors [2]. However, the remission rate of MDD remains relatively low in clinical settings, with an average of approximately 30–40% per month. This low remission rate is primarily attributed to the unclear understanding of the pathological mechanism underlying MDD. In recent years, regarding the pathogenic mechanisms of MDD, in neurobiology, researchers found that neuroinflammation plays a very important role [3]; in neuroimaging, using functional magnetic resonance imaging (fMRI), researchers found that the prefrontal lobe, cerebellum, and brainstem may be involved in the pathological mechanisms of insensitivity to antidepressant drugs in patients with MDD [4]. Although several neuroimaging studies were performed to explore the pathogenesis of depressive disorders, the causal association between depressive disorders and functional (FC) and structural connectivity (SC) of the brain has not yet been revealed [5, 6].

MRI has been widely used to elucidate the neural correlates of clinical symptoms, treatment responses, and disease prognosis in patients with MDD. On the one hand, previous studies discovered that FC within the frontoparietal control systems of patients with MDD was markedly reduced [7], and a multisite sample used to reliably analyze the FC of the brain suggested that the global and node efficiencies of FC local networks decreased at the nodal level in patients with depressive disorders assessed using neuroimaging [8]. On the other hand, some researchers have found that global SC strength was reduced in patients with MDD [9], suggesting that abnormal resting-state brain connectivity is significantly correlated with MDD. FC reflects the interregional similarity in the pattern of time-varying fluctuations in functional brain activation in the task-free state. The resting-state SC of the brain refers to the physical interconnection of brain regions through white matter tracts [10]. FC can be used to address the questions of serial, parallel, local, or total neural processing in cognitive neuroscience and explore the mechanisms of perceptual multisensory integration in depressive disorders [11]. By contrast, SC can be used to evaluate whether white matter structures are damaged in psychiatric disorders, exposing associations between or within the brain regions, such as the normal or pathological functioning of the amygdala [12]. Based on the research on SC or FC, Tissink et al. divided the entire brain into seven best-known resting-state networks (RSNs): the default mode network (DMN), dorsal attention network (DAN), global network (GN), limbic network (LN), somatomotor network (SMN), frontoparietal network (FPN), ventral attention network (VAN), and visual network (VN) [13].

MDD is a complex mental disorder characterized by alterations in various brain networks. The DMN, which comprises brain components distributed in the parietal, temporal, and frontal cortices, plays a crucial role in memory and abstract thought [14]. Intriguingly, studies have consistently shown decreased FC within the DMN among individuals with depressive disorders, particularly those with recurrent depressive disorders [15]. However, the underlying mechanisms of these alterations remain elusive, reflecting an unclear etiology of depressive disorders.

Further complicating this picture, recent research revealed divergent findings in individuals with MDD and a history of childhood trauma. In this subgroup, increased FC in the DMN was observed along with decreased FC within the FPN [16]. These contrasting results highlight the heterogeneity and complexity of depressive disorders and suggest that different etiological factors contribute to distinct neurobiological alterations.

The LN, comprising the amygdala, nucleus accumbens, orbitofrontal gyrus, and subgenual anterior cingulate cortex, plays a vital role in the processing and regulation of emotions. Dysfunction within the LN is consistently implicated in depressive disorders, suggesting emotional processing and regulation deficits as potential contributors to depressive symptomatology [17, 18].

Overall, neurobiological alterations within these brain networks highlight the complexity of depressive disorders. The unclear etiology of depressive disorders emphasizes the need for further research to unravel the underlying mechanisms driving these network dysfunctions. By elucidating the etiological factors and their impact on brain networks, we can advance our understanding of depressive disorders and develop targeted interventions for individuals with depressive symptoms.

However, numerous existing observational neuroimaging studies have not explained the causality between depressive disorders and neuroimaging alterations, and critically addressing the cause and effect is essential to deeply explore the neurobiological mechanisms. A Mendelian randomization (MR) study can be utilized for this purpose. MR uses genetic variation as an instrumental variable to assess causality. It is based on the random assignment of genetic variants associated with the exposure of interest to infer the causal effect of that exposure on a specific outcome. Hence, in the present study, we investigated the effect of brain RSNs on depressive disorders using a bidirectional MR design, in which genetic variants known to be associated with RSNs were used as instrumental variables. These genetic variants were randomly assigned to the population and were not subjected to confounding factors. By analyzing the association between the genetic variant and the outcome variable, causal inferences can be made regarding the effect of exposure.

## Materials and methods

### Summary-level genome-wise association study data for evaluating cerebral RSNs and selecting single-nucleotide polymorphisms

Neuroimaging data from 40,682 volunteers from the UK Biobank were used in this study [19]. The Complex Trait Genetics Lab provided summary-level genome-wise association study (GWAS) data, which were correlated with brain RSNs and obtained from a genetic architecture analysis of participants with European ancestry. Several exclusion criteria were applied to ensure data quality, including non-European ancestry, withdrawn consent, relatedness identified by the UK Biobank, discordant sex, and sexual aneuploidy. Additional details regarding these criteria can be found in a previous study [20]. The assessment of FC and SC in the resting state was based on the study by Yeo [21]. This involved measuring the connectivity using resting-state functional brain imaging (rs-fMRI) and multiband diffusion brain imaging. The obtained data were processed using the structural and functional pipelines of CATO. A rigorous procedure was followed to select the genetic instruments that strongly predicted cerebral RSNs. The independence of the instruments was ensured by considering linkage disequilibrium (LD) ${\text{r}}^{2}$, with a threshold of ${\text{r}}^{2}<0.01$ and < 1 MB proximity from the index variant. However, when a strict GWAS-correlation threshold of $\text{P}<5\times {10}^{-8}$ is applied, the number of significant results would be insufficient for subsequent studies. Therefore, a more relaxed threshold of $\text{P}<5\times {10}^{-5}$, commonly used in previous MR studies, was adopted to include additional single-nucleotide polymorphisms (SNPs) that contributed to RSNs [22].

### Summary-level GWAS data for evaluating depressive disorders and selecting SNPs

Genetic predictors and associations related to depressive disorders were obtained from an updated GWAS conducted by the FinnGen Consortium [23]. The GWAS included 48,847 patients with depressive disorders and 225,483 controls with European ancestry. FinnGen defines depressive disorders based on the International Classification of Diseases diagnosis codes, and the GWAS data were adjusted for age, sex, and genotyping batch. Heterogeneity across the meta-analyses of previous studies was evaluated using I2 statistics and the Q test, suggesting minimal heterogeneity among the included studies (I2 < 25%, Q statistic *p*-value > 0.05).

To represent depressive disorders in the MR analysis, genetic instruments were selected based on specific criteria. These criteria included a GWAS-correlated *P*-value of $5\times {10}^{-8}$, an LD of ${\text{r}}^{2}<0.01$, and a proximity of < 1 MB from the index variant. Following the selection process, 20 useful SNPs were identified and utilized in the MR analysis. Detailed information regarding these index SNPs can be found in Supplementary Table S1.

To assess the potential bias caused by sample overlap, we used a web tool developed by Burgess et al. An analysis indicated a negligible overlap of individuals between the exposure and outcome studies, with less than 1% estimated overlap, thereby minimizing the risk of sample overlap bias[available at https://sb452.shinyapps.io/overlap/] [24]. The results of the analysis indicated that the level of bias was negligible at less than 1%. An analysis indicated a negligible overlap of individuals between the exposure and outcome studies, with less than 1% estimated overlap, thereby minimizing the risk of sample overlap bias.

Genetic instruments with palindromic sequences were removed to ensure that the selected SNPs were aligned in the same direction for exposure and outcome. Unfortunately, GWASs of the exposures did not provide allele frequency information for these SNPs.

Summary information regarding the SNPs associated with the 16 traits is provided in Supplementary Tables S2-17.

### MR analyses

MR studies commonly use three methods to address variant sensitivity and potential pleiotropic effects: random-effect inverse-variance weighted (IVW), MR-Egger, and weighted median [25]. The IVW method is the primary approach and assumes that genetic instruments affect the outcome only through the exposure of interest, without being influenced by alternative pathways. This provides the main outcome estimates. However, to enhance the robustness of IVW estimates, MR-Egger and weighted median methods are employed. These methods complement the results in a broader range of scenarios, although they are less efficient and yield wider confidence intervals. The weighted median method allows for the inclusion of invalid instruments as long as at least half of the instruments used in the MR analysis are valid [26]. By contrast, MR-Egger allows for the inclusion of genetic variants with pleiotropic effects but requires that these pleiotropic effects are independent of the variant-exposure association [27]. In MR studies, the presence of outliers in selected SNPs representing exposures can be identified using the MR-PRESSO method. These outliers can be removed, and MR-PRESSO can be used to test for significant differences in the causal estimates before and after outlier correction [28]. If the estimates obtained from these MR methods are inconsistent, researchers should consider setting a tighter instrument *P*-value threshold to improve the reliability of the results [29]. For significant estimates, directional pleiotropy can be assessed using the MR-Egger intercept test, with a *P*-value below 0.05 indicating the presence of directional pleiotropy [30]. Additionally, a funnel plot can be used to visually evaluate possible directional pleiotropy, and Cochran’s Q test can be employed to assess heterogeneity among the included studies.

**Fig. 1:**
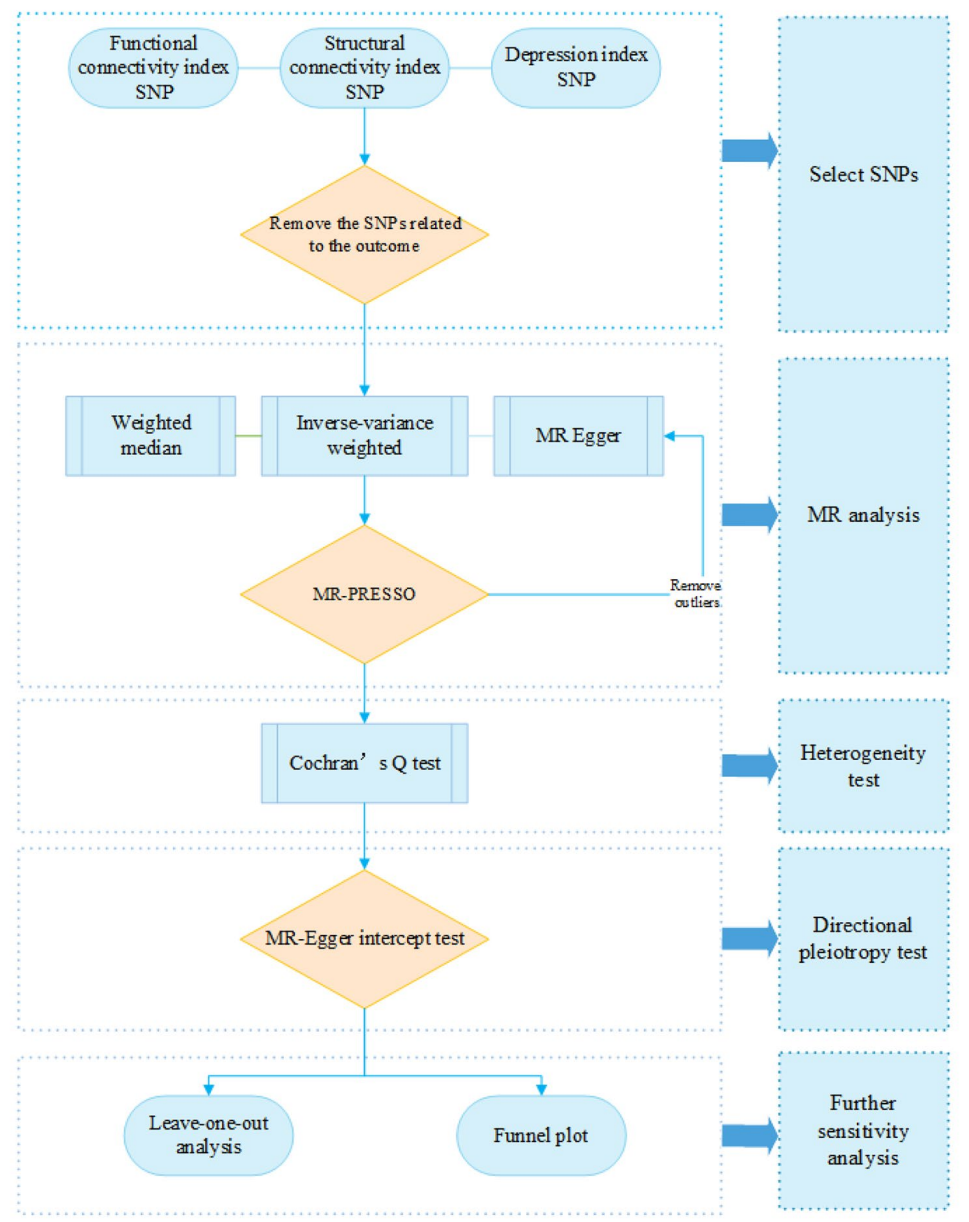
Flow chart of selecting the genetic instruments and completing Mendelian randomization analysis. SNP, single-nucleotide polymorphism; MR, Mendelian randomization; MR-PRESSO(Mendelian Randomization-Pleiotropy RESidual Sum and Outlier)

To examine whether the MR estimate was driven or biased by a single SNP, we performed a leave-one-out analysis. This involved sequentially removing each SNP from the analysis to assess its impact on the overall estimate. The process of conducting the MR study is shown in Fig. 1. To further ensure the robustness and validity of our Mendelian randomization analysis, we calculated the F-statistics for each of the selected genetic instruments to assess their strength. The F-statistic is a measure of the instrumental variable strength, where higher values indicate a stronger instrument. It is calculated using the formula: F = (R² / (1 - R²)) * (*n* - k − 1), where R² is the proportion of variance in the exposure explained by the instrument, *n* is the sample size, and k is the number of instruments. A commonly accepted threshold for a strong instrument is an F-statistic greater than 10, which suggests that the instrument is sufficiently strong to provide reliable MR estimates and minimize the risk of weak instrument bias.

### Statistical analysis

All tests were two-sided, and the Bonferroni-corrected significance threshold was set at $\text{P}<0.003$ (corrected for 16 risks), and $\text{P}<0.05$ was regarded as nominally significant. All the analyses were performed using the packages TwoSampleMR and MR-PRESSO in R (version 4.3.0).

### Ethics

No additional ethical approval was required because the study consisted of the reanalysis of previously collected and published data.

## Results

The F-statistics and quantity of genetic instruments are described in Table 1 in detail. The selected SNPs were all larger than 10, suggesting the absence of weak instrument bias in the present study [31].

**Table 1:** Details of the selected genetic instruments

RSNs	SNPs	F-statistics
FC Default mode network	89	19
FC Dorsal attention network	91	19
FC Global network	80	18
FC Limbic network	82	19
FC Somatomotor network	80	20
FC Frontoparietal network	86	19
FC Ventral attention network	80	18
FC Visual network	61	18
SC Default mode network	85	19
SC Dorsal attention network	102	18
SC Global network	163	23
SC Limbic network	101	18
SC Somatomotor network	103	18
SC Frontoparietal network	163	23
SC Ventral attention network	80	18
SC Visual network	94	20
Depressive disorders	20	38

RSN, resting-state network; SNP, single-nucleotide polymorphism; FC, functional connectivity; SC, structural connectivity

**Fig. 2:**
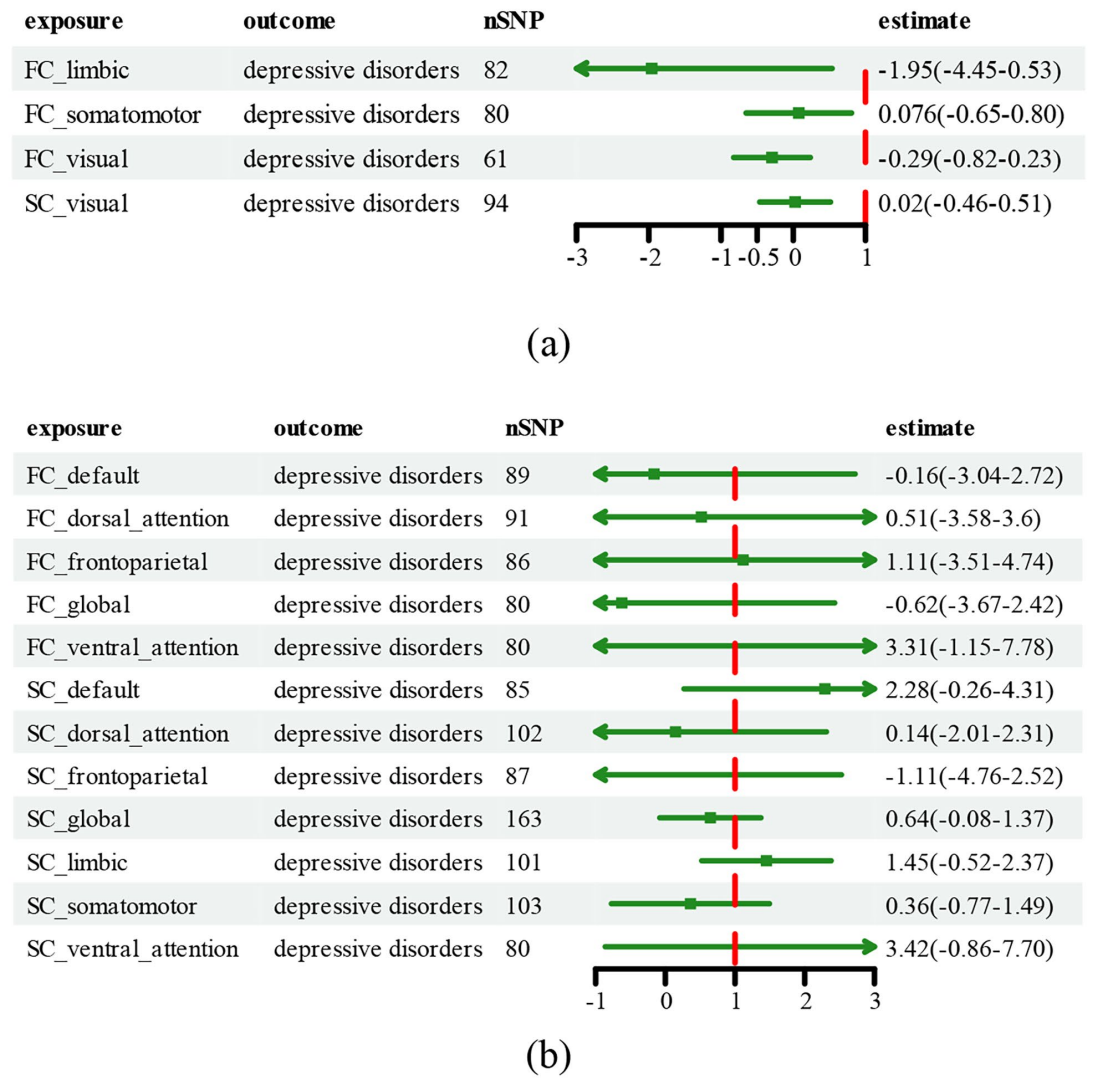
Forest plot of the Mendelian randomization study on resting-state brain networks regarding depressive disorders. Part (**a**) of Figure 3 illustrates the forest plots for the functional connectivity (FC) of the limbic network, somatomotor network, and visual network, along with the structural connectivity (SC) of the visual network. Conversely, part (**b**) of Figure 3 extends our analysis to encompass the remaining networks not covered in part (**a**). This section offers a comprehensive overview of our findings across the broader spectrum of brain networks involved in depressive disorders. The SNPs are selected with a *P*-value of less than 5×10^−5^. After correction for multiple comparisons, a *P*-value of <0.003 is considered significant. The estimates included odds ratio (OR) and 95% confidence interval (CIs), which represented the change in the OR of depressive disorders per one standard deviation (SD) increase in the resting-state network level SNP, single-nucleotide polymorphism; FC, functional connectivity; SC, structural connectivity; FC_default: default mode network (DMN) of FC; FC_dorsal_attention: dorsal attention network of FC; FC_frontoparietal: frontoparietal network of FC; FC_global: global network of FC; FC_limbic: limbic network of FC; FC_somatomotor: somatomotor network of FC; FC_ventral_attention: ventral attention network of FC; FC_visual: visual network of FC; SC_default: default mode network of SC; SC_dorsal_attention: dorsal attention network of SC; SC_frontoparietal: frontoparietal network of SC; SC_global: global network of SC; SC_limbic: limbic network of SC; SC_somatomotor: somatomotor network of SC; SC_ventral_attention: ventral attention network of SC; SC_visual: visual network of SC

In addition, 20 SNPs strongly predicted depressive disorders to be utilized in the reverse estimates, and the results did not suggest a significant effect of depressive disorders on RSNs after correction for multiple testing ($\text{P}<0.003$), especially the causal effect of depressive disorders on SC LN ($\text{O}\text{R}=1.0025$; $\text{C}\text{I}, 1.0005-1.0046$; $\text{P}=0.012$]. Cochran’s Q test indicated that the heterogeneity in the present study was not significant (Cochran’s Q test–derived $\text{P}>0.05$). There was no sign of horizontal pleiotropy in the association between depressive disorders and SC LN, as measured using MR-Egger ($\text{i}\text{n}\text{t}\text{e}\text{r}\text{c}\text{e}\text{p}\text{t}=0.003$; $\text{S}\text{E}=0.004$; $\text{P}=0.432$). The resulting forest plots are shown in Fig. 2. Furthermore, the *P*-value for the global test was 0.32 in the MR-PRESSO test, in which no outlier needed to be removed. Furthermore, we did not find a single SNP that strongly violated the overall effect of RSN on depressive disorders in the leave-one-out sensitivity.

**Fig. 3:**
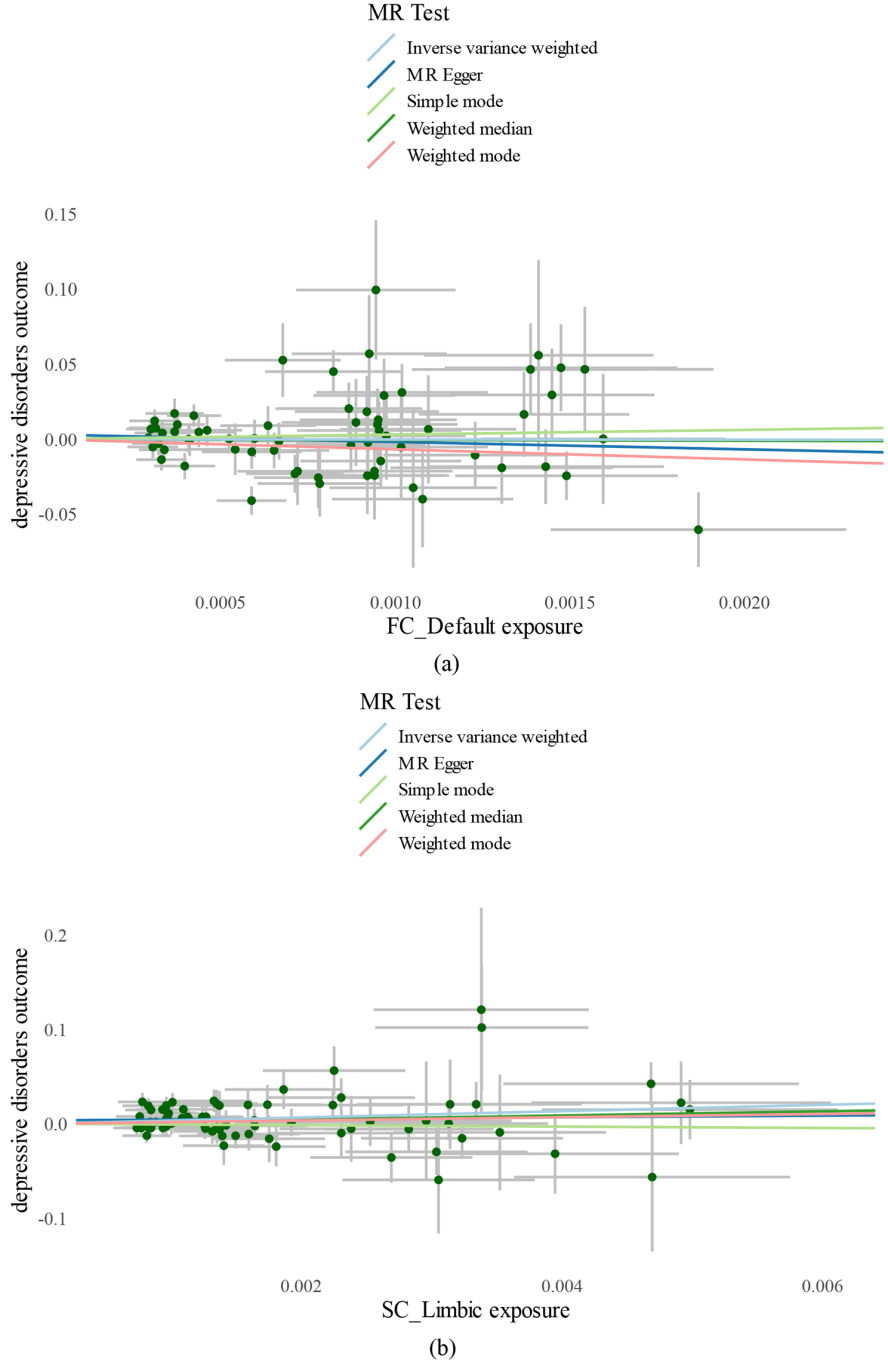
Scatterplot of the result for the effect of FC DMN and SC LN on depressive disorders. MR, Mendelian Randomization; SNP, single-nucleotide polymorphism. Part (**a**) of the figure shows the scatterplot of FC DMN and part (**b**) shows the scatterplot of SC LN Each dot on the graph represents a SNP locus, the horizontal coordinate is the effect of SNP on exposure (FC DMN, SC LN), the vertical coordinate is the effect of the SNP on the outcome (depressive disorders), and the colored line indicates the MR fitting results

**Fig. 4:**
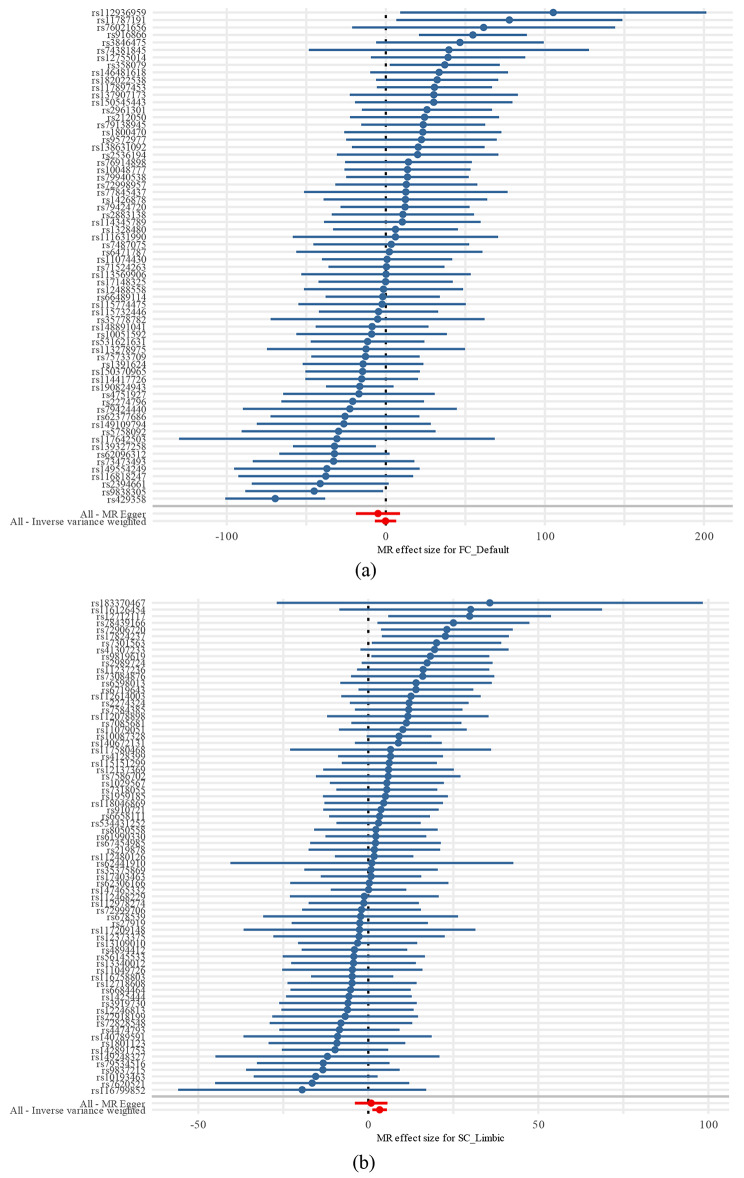
Each horizontal solid line in the forest plot above reflects the results of individual SNPs estimated using the Wald ratio method. MR, Mendelian Randomization; SNP, single-nucleotide polymorphism. Part (**a**) of the figure shows the forest plot of the SNPs of FC DMN and part (**b**) shows the forest plot of the SNPs SC LN. Some solid lines are entirely to the left of 0, indicating that the result estimated from this SNP is that increased exposure reduces the risk of depressive disorders; some solid lines are entirely to the right of 0, indicating that the result estimated from this SNP is that increased exposure elevates the risk of depressive disorders. Those crossing 0 indicate that the result is not significant. To get a reasonable result is to look at the results together, and that’s the bottom red line, which reflects the estimation of the effect of exposure on outcome under the IVW method. The red line for FC DMN ranges across 0, indicating that it has a nonsignificant causal effect on depressive disorders, whereas the red line for SC LN totally lies to the right of 0, indicating that as the intensity of SC LN increases, the risk of developing depressive disorders increases

In the random-effect IVW estimates, we found that the genetically predicted SC LN was potentially associated with a decreased risk of depressive disorders (odds ratio [OR], 28.21; 95% confidence interval [CI], 3.32–239.54; *P* = 0.002); this result is consistent with the results of weighted median and MR-Egger, suggesting that this causal effect was robust, more information can be seen in the scatterplot Fig. 3 for FC DMN and SC LN, More detailed scatterplots of other results of IVW, weighted median and MR-Egger are available in the Supplementary Fig.S1-S30. It is possible to visualize the results of individual SNPs of FC DMN and SC LN estimated using the Wald ratio method in Fig. 4, more attention needs to be paid to the combined results of each SNP effect, which is the red line at the very end of the graph corresponding to IVW, where it can be clearly seen that the causal effect of FC DMN on depressive disorders was not significant and the higher the intensity of the SC LN, the higher the risk of developing depressive disorders. However, heterogeneity was not observed in the Cochran’s Q test–derived *P*-value of 0.40 for MR-Egger, and the *P*-value of 0.39 for IVW.MR-PRESSO presented a similar result (*P*-value in the global heterogeneity test was 0.40). No outliers were detected. Furthermore, there was no evidence of a significant intercept (intercept = 0.003; SE = 0.003; *P* = 0.257); consequently, there was no directional pleiotropy. We did not find a single SNP that strongly violated the overall effect of the RSN on depressive disorders in the leave-one-out sensitivity analysis.

No association was found between genetically determined FC and depressive disorders; more specific negative results are provided in the Supplementary Material. When FC DMN, depressive disorders, SC SMN, and SC VAN were set as exposure variables, some outliers were removed; however, after removing the outliers, the results remained nonsignificant.

## Discussion

To the best of our knowledge, this is the first MRI study to use a bidirectional design to explore the cause-and-effect relationship between brain RSNs and depressive disorders. This design allowed us to examine the possibility of reverse causation. In addition, we employed several analyses to ensure the robustness of the results. Furthermore, the GWAS data used in this study have been updated, enabling us to track the progression of brain RSNs over time. Considering the findings from our analyses, there appears to be a discernible association between an increase in SC LN strength and a higher risk of developing depressive disorders under specific conditions identified in our study. This preliminary observation suggests a potential pathway through which SC LN alterations may contribute to the pathophysiology of depressive disorders. However, it is imperative to underscore that these findings are context-dependent and may not be universally applicable across different populations or conditions. In summary, this bidirectional MR study suggests that SC LN has a causal effect on depressive disorders. This causal relationship underscores the significance of examining neural connectivity and its impact on mental health conditions. However, the results did not indicate a causal connection between FC and depressive disorders. We found that SC LN with increased white matter integrity might be the etiological network of depressive disorders, which is consistent with that reported in previous research [32]. Several previous studies have also found that white matter microstructures are disrupted in the brains of patients with depressive disorders [33], which may be related to differences in white matter alterations in specific regions of the brain in these patients, as confirmed in previous studies that observed the white matter of the depressed brain using a segmentation methodology [34]. Based on these previous studies, we hypothesized several potential mechanisms by which SC LN promotes the development of depressive disorders. The first hypothesis was that there is an alteration in the neuronal cell density, as in Parkinson’s disease, wherein neuronal loss would be expected to result in a decrease in white matter integrity; however, a reduction in the glial density owing to an increase in the axon-to-cell-body ratio may increase white matter integrity, which could also explain the damage that occurs in a portion of the white matter [35]. Second, some researchers observed a reduction in gray matter volume in the brain in patients with depressive disorders, and in addition, depressive disorders showed to be associated with gray matter volume in an MR study [36, 37]; therefore, we speculate that there is a compensatory mechanism in the structural connectivity of these patients. For example, on trait anxiety, Montag et al. proposed the hypothesis that enhanced white matter integrity in the temporal lobe compensates for the reduction in hippocampal gray matter [38], and a recent MR study on brain structure and anxiety disorders suggests that reductions in gray matter volume in the right anterior superior temporal gyrus have a direct impact on anxiety disorders, and that most psychiatric disorders may share common underlying mechanisms [39, 40]. Third, increased white matter integrity may not be related to the core symptoms of depressive disorders but is strongly associated with a subgroup of depressive disorders, such as in markedly anxious patients with depressive disorders in whom such brain imaging changes are evident [35]. Wen et al. reported that two types of depressive disorders can be observed in older adults: (1) the white matter integrity of patients with depressive disorders does not significantly differ from that of normal controls and (2) the white matter integrity of patients with depressive disorders is extensively impaired. Both types of depressive disorders are genetically linked and support our belief that SC LN may be etiologically linked to a particular subgroup of depressive disorders [41]. Fourth, some researchers have found that enhanced white matter integrity can be observed in male patients, not in female patients, with depressive disorders [38]. This suggests that androgens may have a maintenance or enhancement effect on white matter integrity, which was corroborated in a study of androgen deprivation therapy in patients with prostate cancer [42]. Finally, several studies have focused on the effects of medications on the brain structure [43]. In a recent study, researchers found that, although the cortical thickness was altered in patients with depressive disorders taking olanzapine versus sertraline for 2 weeks, there was no significant change in white matter integrity; therefore, the results of the present study cannot be explained entirely by the drug effect [44]. It is the SC LN rather than other SC networks that demonstrated a causal relationship with depressive disorders in this study. Because relatively little research has been conducted on SC LN, we hypothesized that this may be attributed to the presence of brain regions important for the onset of depressive disorders, such as the amygdala and orbital frontal lobes. Previous research has confirmed the importance of prefrontal brain regions in the etiology of depressive disorders, and the integrity of the amygdala is markedly disrupted in elderly patients with depressive disorders [45, 46].

Recent studies have reported widespread alterations in FC in patients with depressive disorders. For instance, Pan et al. revealed that ventral striatal FC could predict the risk of depressive disorders, which contrasts with the nonsignificant consequences observed in the present study [47]. These inconsistencies might be attributed to alterations in SC affecting FC or to FC being more closely associated with symptom severity rather than the development of depressive disorders. Several studies have demonstrated correlations between FC and specific symptoms such as rumination or appetite changes [5, 44]. Furthermore, FC in depressive disorders can vary depending on several factors such as the age of onset, medication status, depressive symptom patterns, illness duration, and treatment response [10]. Short-term and learning-induced changes in FC can influence these results, suggesting that FC may not be a direct factor in the etiology of depressive disorders [48]. Moreover, heterogeneity in sample sizes across studies may have contributed to the variability in the results. Although neuroinflammation has been proposed as a potential mechanism underlying altered FC in depressive disorders, further exploration is needed. Nonetheless, FC can be used not only to characterize depressive disorders but also as a target for drug interventions to improve the symptoms [49, 50].

Our study has several limitations. First, MR relies on specific assumptions and may be influenced by factors such as instrument selection and population characteristics. Second, the quality of this study depended on the original GWAS data. Third, the causality between brain RSNs and depressive disorders in other populations remains unknown because the enrolled patients were predominantly of European descent. Additionally, we observed a relatively large OR value, a similar issue also noted in another article utilizing the same RSNs database [51]. This could be due to measurement errors within the database, and it may suggest a weaker association between genes and depressive disorders. While this observation lends support to the theory that depressive disorders is a trait influenced by a polygenic inheritance model, we advise readers to interpret our results with caution [52]. Finally, although we reported the causal relationships, we did not extensively explore the potential neurobiological mechanisms underlying these relationships.

The results of this study may direct the clinical or basic research to pay more attention to SC LN in patients in the early stages of depressive disorders or in those who do not meet the diagnostic criteria for depressive disorders but have some depressive symptoms as well as to test the network in a randomized controlled trial to explore effective interventions in the future.

## Conclusion

We conducted this comprehensive MR study to provide evidence that the increased strength level of SC LN is casually associated with an increased risk of depressive disorders. Moreover, we observed no significant results in reverse MR estimates. Relative risk estimates were translated into absolute risk reductions for a meaningful time period, providing practical insights into the potential impact of modifying exposure levels on depressive disorders outcomes. In future research on FC and SC, we need to pay more attention to SC LN not only to discover the mechanism of disease initiation and prevent the disease but also to provide effective treatment.

### Electronic supplementary material

Below is the link to the electronic supplementary material.

**Supplementary Material 1:** The selected SNPs of SC DMN

**Supplementary Material 2:** The selected SNPs of SC FPN

**Supplementary Material 3:** The selected SNPs of FC GN

**Supplementary Material 4:** The selected SNPs of SC DAN

**Supplementary Material 5:** The selected SNPs of depressive disorders

**Supplementary Material 6:** The selected SNPs of FC DAN

**Supplementary Material 7:** The selected SNPs of FC DMN

**Supplementary Material 8:** The selected SNPs of SC VN

**Supplementary Material 9:** The selected SNPs of FC VN

**Supplementary Material 10:** The selected SNPs of FC SMN.

**Supplementary Material 11:** The selected SNPs of FC VAN

**Supplementary Material 12:** The selected SNPs of SC GN

**Supplementary Material 13:** The selected SNPs of SC SMN

**Supplementary Material 14:** The selected SNPs of SC LN

**Supplementary Material 15:** The selected SNPs of FC LN

**Supplementary Material 16:** The selected SNPs of SC VAN

**Supplementary Material 17:** STROBE-MR checklist of recommended items to address in reports of Mendelian randomization studies

**Supplementary Material 18:** The scatterplots of specific negative results

## Data Availability

Data availability: The GWAS summary data of CTG can be obtained from https://ctg.cncr.nl/software/summary_statistics; the GWAS summary data of FinnGen(R9) be obtained from https://www.finngen.fi/en/access_results.

## Abbreviations

FC: Functional connectivity
SC: Structural connectivity
RSN: Resting-state network
rs-fMRI: Resting-state functional magnetic resonance imaging
DMN: Default mode network
DAN: Dorsal attention network
VN: Visual network
LN: Limbic network
SMN: Somatomotor network
VAN: Ventral attention network
FPN: Frontoparietal network
GN: Global network
OR: Odds ratio
CI: Confidence interval
GWAS: Genome-wide association study
MR: Mendelian randomization
MR-PRESSO: Mendelian Randomization-Pleiotropy RESidual Sum and Outlier
MDD: Major depressive disorder
LD: Linkage disequilibrium
SNP: Single-nucleotide polymorphism
IVW: Inverse-variance weighted
