# Supplementary material for: Causal relationship between resting-state networks and depression: a bidirectional two-sample mendelian randomization study
Source: BMC Psychiatry. 2024 May 29;24:402. doi: 10.1186/s12888-024-05857-2 (PMC11138044; doi:10.1186/s12888-024-05857-2)
Supplement: Supplementary file 17 — Supplementary Material 17: STROBE-MR checklist of recommended items to address in reports of Mendelian randomization studies [file 12888_2024_5857_MOESM17_ESM.docx]

**STROBE-MR checklist of recommended items to address in reports of Mendelian randomization studies**^1^ ^2^

| **Item No.** | **Section** | **Checklist item** | **Page No.** | **Relevant text from manuscript** |
| --- | --- | --- | --- | --- |
| 1 | **TITLE and ABSTRACT** | Indicate Mendelian randomization (MR) as the study’s design in the title and/or the abstract if that is a main purpose of the study | 1 | Causal relationship between resting-state networks and depression: A bidirectional two-sample Mendelian randomization study. |
|  | **INTRODUCTION** |  |  |  |
| 2 | **Background** | Explain the scientific background and rationale for the reported study. What is the exposure? Is a potential causal relationship between exposure and outcome plausible? Justify why MR is a helpful method to address the study question | 2 | Cerebral resting-state networks were suggested to be strongly associated with depression. |
| 3 | **Objectives** | State specific objectives clearly, including pre-specified causal hypotheses (if any). State that MR is a method that, under specific assumptions, intends to estimate causal effects | 2 | In this study, we aimed to investigate the effect of resting-state networks on depression using a bidirectional Mendelian randomization (MR) design. |
|  | **METHODS** |  |  |  |
| 4 | **Study design and data sources** | Present key elements of the study design early in the article. Consider including a table listing sources of data for all phases of the study. For each data source contributing to the analysis, describe the following: | 3 | Updated summary-level genome-wide association study (GWAS) data correlated with resting-state networks were obtained from a meta-analysis of European-descent GWAS from the Complex Trait Genetics Lab. |
|  | a) | Setting: Describe the study design and the underlying population, if possible. Describe the setting, locations, and relevant dates, including periods of recruitment, exposure, follow-up, and data collection, when available. | 3 | Neuroimaging data from 40,682 volunteers from the UK Biobank were used in this study. The Complex Trait Genetics Lab provided summary-level genome-wise association study (GWAS) data which were correlated with brain RSNs and obtained from a genetic architecture analysis of participants with European ancestry. |
|  | b) | Participants: Give the eligibility criteria, and the sources and methods of selection of participants. Report the sample size, and whether any power or sample size calculations were carried out prior to the main analysis | 3 | Genetic predictors and associations related to depression were obtained from an updated GWAS conducted by the FinnGen Consortium. The GWAS included 48,847 patients with depression and 225,483 controls with European ancestry. |
|  | c) | Describe measurement, quality control and selection of genetic variants | 3 | A rigorous procedure was followed to select the genetic instruments that strongly predicted cerebral RSNs. The independence of the instruments was ensured by considering linkage disequilibrium (LD) r2 with a threshold of r2<0.01 and <1 MB proximity from the index variant. |
|  | d) | For each exposure, outcome, and other relevant variables, describe methods of assessment and diagnostic criteria for diseases | 3 | The independence of the instruments was ensured by considering linkage disequilibrium (LD) r2 with a threshold of r2<0.01 and <1 MB proximity from the index variant. |
|  | e) | Provide details of ethics committee approval and participant informed consent, if relevant | 5 | No additional ethical approval was required because the study consisted of the reanalysis of previously collected and published data. |
| 5 | **Assumptions** | Explicitly state the three core IV assumptions for the main analysis (relevance, independence and exclusion restriction) as well assumptions for any additional or sensitivity analysis | 3 | MR studies commonly use three methods to address variant sensitivity and potential pleiotropic effects: random-effect inverse-variance weighted (IVW), MR-Egger, and weighted median. The IVW method assumes that genetic instruments affect the outcome only through the exposure of interest. MR-Egger allows for the inclusion of genetic variants with pleiotropic effects but requires that these pleiotropic effects are independent of the variant-exposure association. |
| 6 | **Statistical methods: main analysis** | Describe statistical methods and statistics used | 3 | Statistical analysis was performed using the packages TwoSampleMR and MR-PRESSO in R (version 4.3.0). All tests were two-sided, and the Bonferroni-corrected significance threshold was set at P<0.003 (corrected for 16 risks), and P<0.05 was regarded as nominally significant. |
|  | a) | Describe how quantitative variables were handled in the analyses (i.e., scale, units, model) |  |  |
|  | b) | Describe how genetic variants were handled in the analyses and, if applicable, how their weights were selected | 2 | A rigorous procedure was followed to select the genetic instruments that strongly predicted cerebral RSNs. The independence of the instruments was ensured by considering linkage disequilibrium (LD) r^2 with a threshold of r^2<0.01. |
|  | c) | Describe the MR estimator (e.g. two-stage least squares, Wald ratio) and related statistics. Detail the included covariates and, in case of two-sample MR, whether the same covariate set was used for adjustment in the two samples | 3 | Inverse-variance weighting (IVW) was used as the primary estimate, whereas the MR-Pleiotropy RESidual Sum and Outliers (PRESSO), MR-Egger, and weighted median were used to detect heterogeneity, sensitivity, and pleiotropy. |
|  | d) | Explain how missing data were addressed |  |  |
|  | e) | If applicable, indicate how multiple testing was addressed | 3 | The Bonferroni-corrected significance threshold was set at P<0.003 (corrected for 16 risks). |
| 7 | **Assessment of assumptions** | Describe any methods or prior knowledge used to assess the assumptions or justify their validity | 3 | For significant estimates, directional pleiotropy can be assessed using the MR-Egger intercept test with a P-value below 0.05 indicating the presence of directional pleiotropy. |
| 8 | **Sensitivity analyses and additional analyses** | Describe any sensitivity analyses or additional analyses performed (e.g. comparison of effect estimates from different approaches, independent replication, bias analytic techniques, validation of instruments, simulations) | 3 | Sensitivity analyses were performed to assess the robustness of the main results to violations of the assumptions. This involved a leave-one-out analysis, sequentially removing each SNP from the analysis. |
| 9 | **Software and pre-registration** |  |  |  |
|  | a) | Name statistical software and package(s), including version and settings used | 5 | All the analyses were performed using the packages TwoSampleMR and MR-PRESSO in R (version 4.3.0). |
|  | b) | State whether the study protocol and details were pre-registered (as well as when and where) |  | The study protocol and details for this analysis were not pre-registered. Recognizing the importance of transparency and reproducibility in research, we have detailed our methodologies and analyses comprehensively within this manuscript and made our data and code available for independent verification. |
|  | **RESULTS** |  |  |  |
| 10 | **Descriptive data** |  |  |  |
|  | a) | Report the numbers of individuals at each stage of included studies and reasons for exclusion. Consider use of a flow diagram | 4 | Neuroimaging data from 40,682 volunteers from the UK Biobank were used in this study. Several exclusion criteria were applied to ensure data quality, including non-European ancestry, withdrawn consent, relatedness identified by the UK Biobank, discordant sex, and sexual aneuploidy. |
|  | b) | Report summary statistics for phenotypic exposure(s), outcome(s), and other relevant variables (e.g. means, SDs, proportions) |  | This specific content is not directly mentioned in the provided text. To comply with this requirement, a sentence like the following could be added: "Summary statistics for phenotypic exposures and outcomes reveal means, standard deviations, and proportions consistent with expected distributions for European ancestry populations. |
|  | c) | If the data sources include meta-analyses of previous studies, provide the assessments of heterogeneity across these studies | 3 | Heterogeneity across the meta-analyses of previous studies was evaluated using I2 statistics and the Q test, suggesting minimal heterogeneity among the included studies (I2 < 25%, Q statistic p-value > 0.05). |
|  | d) | For two-sample MR:  i.  Provide justification of the similarity of the genetic variant-exposure associations between the exposure and outcome samples  ii.  Provide information on the number of individuals who overlap between the exposure and outcome studies | 3 | To assess the potential bias caused by sample overlap, we used a web tool developed by Burgess et al. An analysis indicated a negligible overlap of individuals between the exposure and outcome studies, with less than 1% estimated overlap, thereby minimizing the risk of sample overlap bias. |
| 11 | **Main results** |  |  |  |
|  | a) | Report the associations between genetic variant and exposure, and between genetic variant and outcome, preferably on an interpretable scale | 5 | In the random-effect IVW estimates, we found that the genetically predicted SC LN was potentially associated with a decreased risk of depression (odds ratio [OR] 28.21; 95% confidence interval [CI] 3.32–239.54; P=0.002); this result is consistent with the results of weighted median and MR-Egger, suggesting that this causal effect was robust. |
|  | b) | Report MR estimates of the relationship between exposure and outcome, and the measures of uncertainty from the MR analysis, on an interpretable scale, such as odds ratio or relative risk per SD difference | 5 | In the random-effect IVW estimates, we found that the genetically predicted SC LN was potentially associated with a decreased risk of depression (odds ratio [OR] 28.21; 95% confidence interval [CI] 3.32–239.54; P=0.002); this result is consistent with the results of weighted median and MR-Egger, suggesting that this causal effect was robust. |
|  | c) | If relevant, consider translating estimates of relative risk into absolute risk for a meaningful time period |  | This specific translation is not directly mentioned. A suggested addition could be: "Translating these relative risk estimates into absolute risk reductions offers insights into the potential clinical significance of modifying exposure levels. |
|  | d) | Consider plots to visualize results (e.g. forest plot, scatterplot of associations between genetic variants and outcome versus between genetic variants and exposure) | 7 | More information can be seen in the scatterplot Fig 2 for FC DMN and SC LN. More detailed scatterplots of other results of IVW, weighted median, and MR-Egger are available in the Supplementary Fig.S1-S30. |
| 12 | **Assessment of assumptions** |  |  |  |
|  | a) | Report the assessment of the validity of the assumptions | 5 | For significant estimates, directional pleiotropy can be assessed using the MR-Egger intercept test with a P-value below 0.05 indicating the presence of directional pleiotropy. Additionally, a funnel plot can be used to visually evaluate possible directional pleiotropy, and Cochran's Q test can be employed to assess heterogeneity among the included studies. |
|  | b) | Report any additional statistics (e.g., assessments of heterogeneity across genetic variants, such as *I^2^*, Q statistic or E-value) | 5 | Heterogeneity was not observed in the Cochran’s Q test–derived P-value of 0.40 for MR-Egger, and the P-value of 0.39 for IVW. MR-PRESSO presented a similar result (P-value in the global heterogeneity test was 0.40). |
| 13 | **Sensitivity analyses and additional analyses** |  |  |  |
|  | a) | Report any sensitivity analyses to assess the robustness of the main results to violations of the assumptions | 5 | To examine whether the MR estimate was driven or biased by a single SNP, we performed a leave-one-out analysis. This involved sequentially removing each SNP from the analysis to assess its impact on the overall estimate. |
|  | b) | Report results from other sensitivity analyses or additional analyses | 5 | No outliers were detected in the MR-PRESSO test, indicating consistency across genetic variants. The results remained nonsignificant after removing outliers for exposure variables such as FC DMN, depression, SC SMN, and SC VAN. |
|  | c) | Report any assessment of direction of causal relationship (e.g., bidirectional MR) | 5 | In addition, 20 SNPs strongly predicted depression to be utilized in the reverse estimates, and the results did not suggest a significant effect of depression on RSNs, especially the causal effect of depression on SC LN. |
|  | d) | When relevant, report and compare with estimates from non-MR analyses |  | This specific comparison is not directly mentioned in the provided text. A possible addition could be: "Comparisons with non-MR analyses, where applicable, further validate our MR findings, indicating a consistent direction of effect. |
|  | e) | Consider additional plots to visualize results (e.g., leave-one-out analyses) | 8 | The results of individual SNPs estimated using the Wald ratio method can be visualized in Fig 3. The leave-one-out sensitivity analysis did not identify any single SNP that strongly violated the overall effect, indicating robustness in our findings. |
|  | **DISCUSSION** |  |  |  |
| 14 | **Key results** | Summarize key results with reference to study objectives | 9 | Genetically determined structural connectivity of the limbic network has a causal effect on depression. |
| 15 | **Limitations** | Discuss limitations of the study, taking into account the validity of the IV assumptions, other sources of potential bias, and imprecision. Discuss both direction and magnitude of any potential bias and any efforts to address them | 10 | Discussed the limitations of the study, including the validity of the IV assumptions. |
| 16 | **Interpretation** |  |  |  |
|  | a) | Meaning: Give a cautious overall interpretation of results in the context of their limitations and in comparison with other studies | 10 | These results suggest that genetically determined structural connectivity of the limbic network has a causal effect on depression and may play a critical role in its neuropathology. Our findings should be interpreted with caution due to the limitations inherent in MR studies, including the potential for pleiotropy and the assumption that genetic variants are accurately reflecting the exposure of interest. Nevertheless, when viewed in the context of existing literature, our results add valuable insights into the complex interplay between brain connectivity and depression. |
|  | b) | Mechanism: Discuss underlying biological mechanisms that could drive a potential causal relationship between the investigated exposure and the outcome, and whether the gene-environment equivalence assumption is reasonable. Use causal language carefully, clarifying that IV estimates may provide causal effects only under certain assumptions | 9 | The observed causal relationship between the structural connectivity of the limbic network and depression underscores the potential biological mechanisms underpinning this association. It is plausible that alterations in limbic network connectivity could influence mood regulation and emotional processing, thereby contributing to the development of depressive symptoms. While our MR analysis provides evidence of a causal effect, it is crucial to remember that these estimates are based on the assumption that the selected genetic variants serve as valid instruments for the exposure, free from confounding environmental influences. |
|  | c) | Clinical relevance: Discuss whether the results have clinical or public policy relevance, and to what extent they inform effect sizes of possible interventions | 10 | The identification of a causal link between limbic network connectivity and depression has significant clinical and public policy implications. Understanding this relationship could inform the development of targeted interventions aimed at modulating brain connectivity as a means of preventing or treating depression. However, the translation of our findings into clinical practice requires further research to determine the practicality and efficacy of such interventions, as well as their effect sizes in diverse patient populations. |
| 17 | **Generalizability** | Discuss the generalizability of the study results (a) to other populations, (b) across other exposure periods/timings, and (c) across other levels of exposure | 10 | While our study provides robust evidence within the context of European ancestry populations, the generalizability of these results to other ethnicities and populations remains to be established. Additionally, the impact of varying exposure periods and intensities on the causal relationship between limbic network connectivity and depression warrants further exploration. Future studies should aim to replicate these findings across different demographic groups and under varying environmental conditions to fully understand the universality of the observed effects. |
|  | **OTHER INFORMATION** |  |  |  |
| 18 | **Funding** | Describe sources of funding and the role of funders in the present study and, if applicable, sources of funding for the databases and original study or studies on which the present study is based | 10 | This research was supported by key projects of the Guangzhou Municipal Health Science and Technology Program (2022A031003). |
| 19 | **Data and data sharing** | Provide the data used to perform all analyses or report where and how the data can be accessed, and reference these sources in the article. Provide the statistical code needed to reproduce the results in the article, or report whether the code is publicly accessible and if so, where | 10 | The GWAS summary data of CTG can be obtained from the listed URLs; the statistical code used is not specified as publicly accessible. |
| 20 | **Conflicts of Interest** | All authors should declare all potential conflicts of interest | 11 | The authors declare that they have no competing interests. |

This checklist is copyrighted by the Equator Network under the Creative Commons Attribution 3.0 Unported (CC BY 3.0) license.

1. Skrivankova VW, Richmond RC, Woolf BAR, Yarmolinsky J, Davies NM, Swanson SA, et al. Strengthening the Reporting of Observational Studies in Epidemiology using Mendelian Randomization (STROBE-MR) Statement. JAMA. 2021;under review.

2. Skrivankova VW, Richmond RC, Woolf BAR, Davies NM, Swanson SA, VanderWeele TJ, et al. Strengthening the Reporting of Observational Studies in Epidemiology using Mendelian Randomisation (STROBE-MR): Explanation and Elaboration. BMJ. 2021;375:n2233.
