# Supplementary material for: Causal relationship between resting-state networks and depression: a bidirectional two-sample mendelian randomization study
Source: BMC Psychiatry. 2024 May 29;24:402. doi: 10.1186/s12888-024-05857-2 (PMC11138044; doi:10.1186/s12888-024-05857-2)

Fig.S1


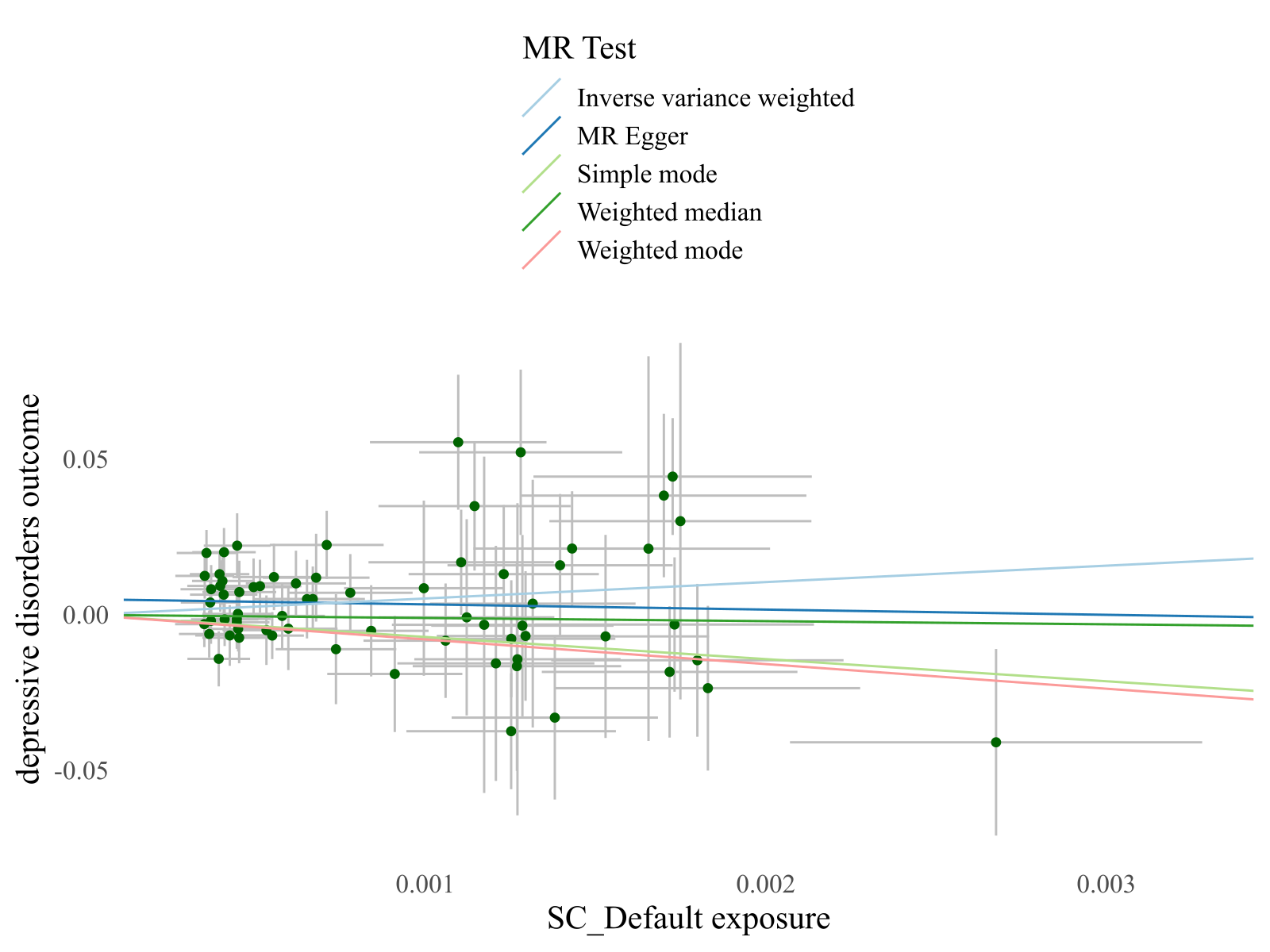


Fig.S2


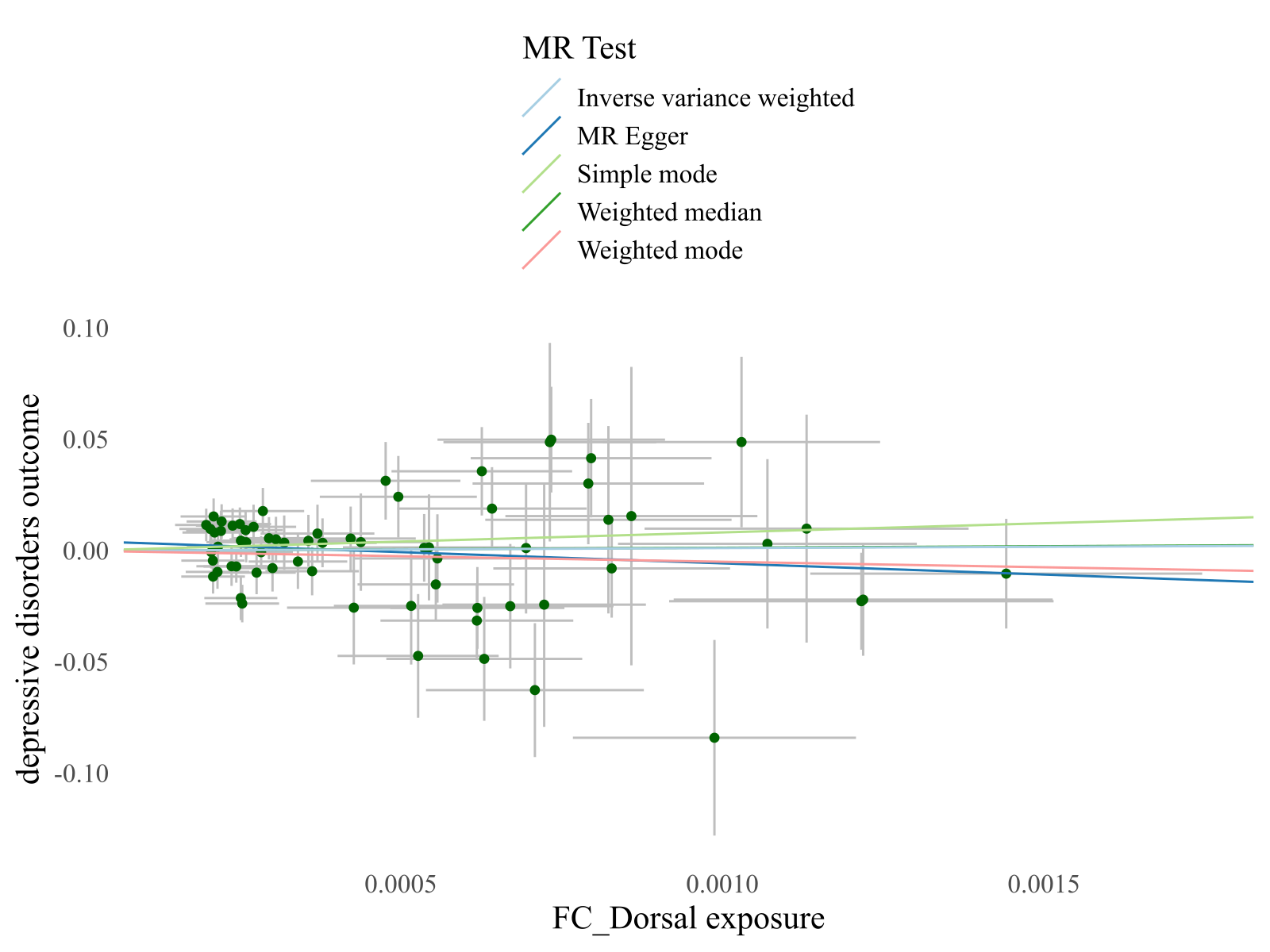


Fig.S3


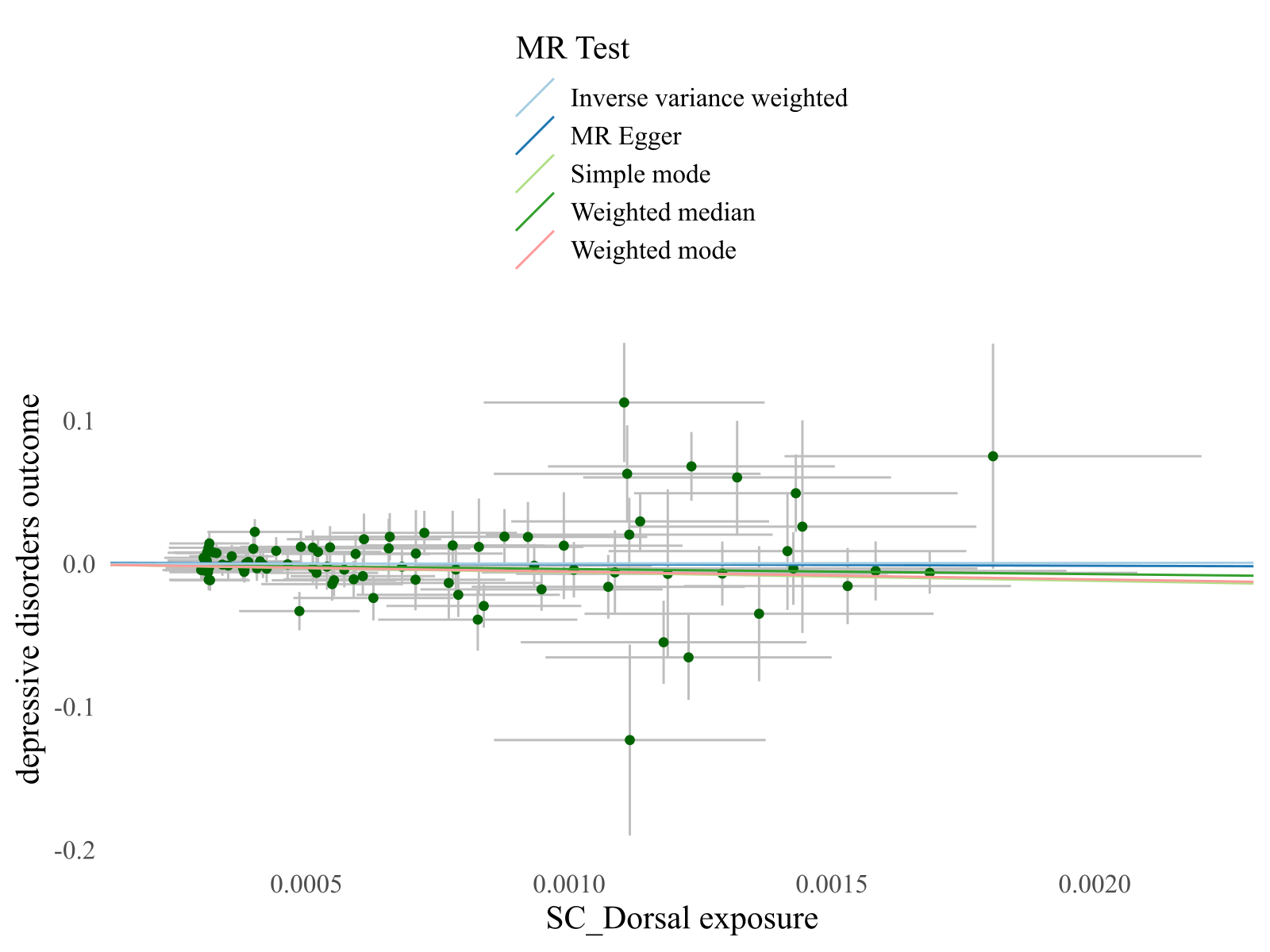


Fig.S4


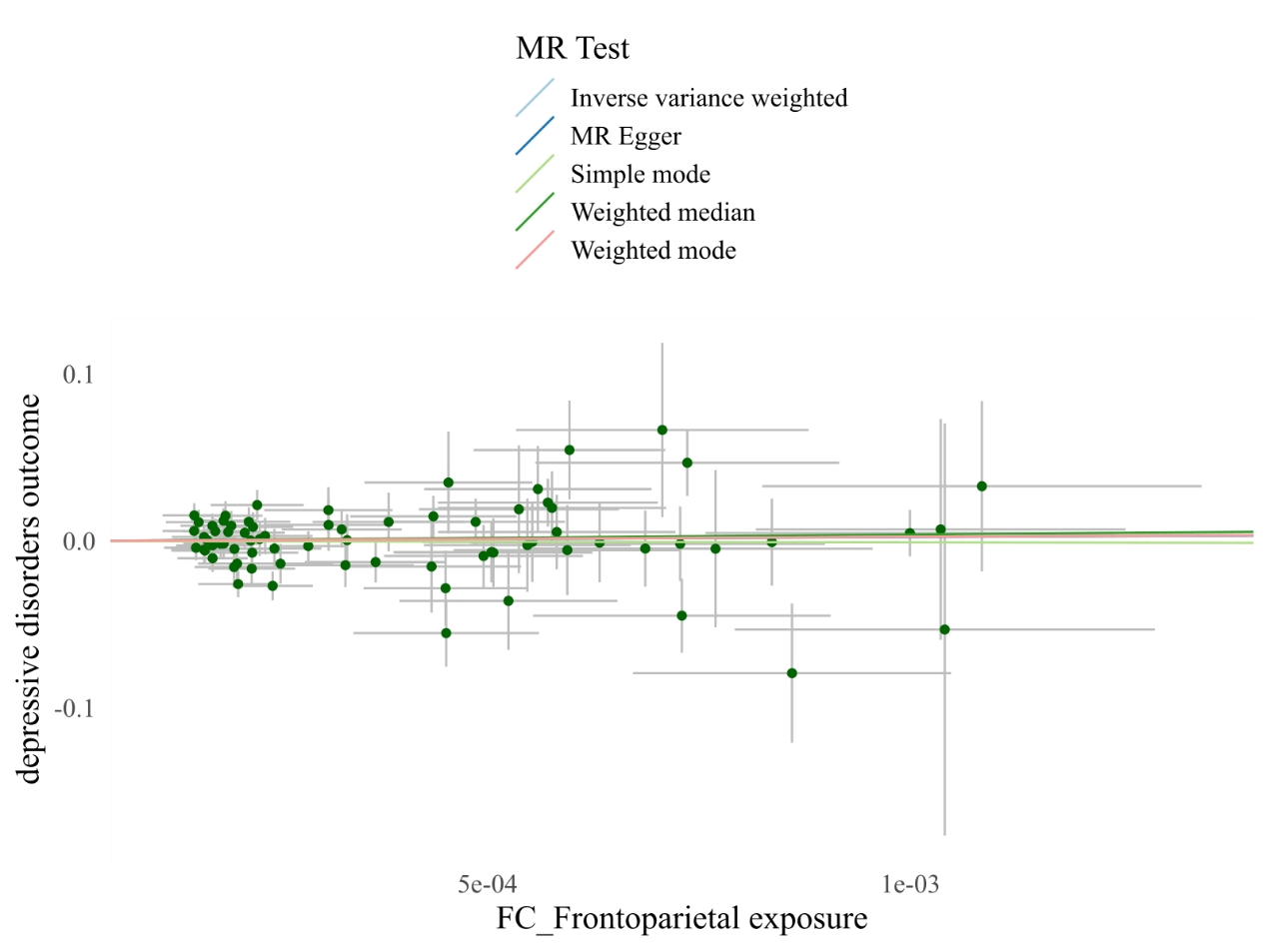


Fig.S5


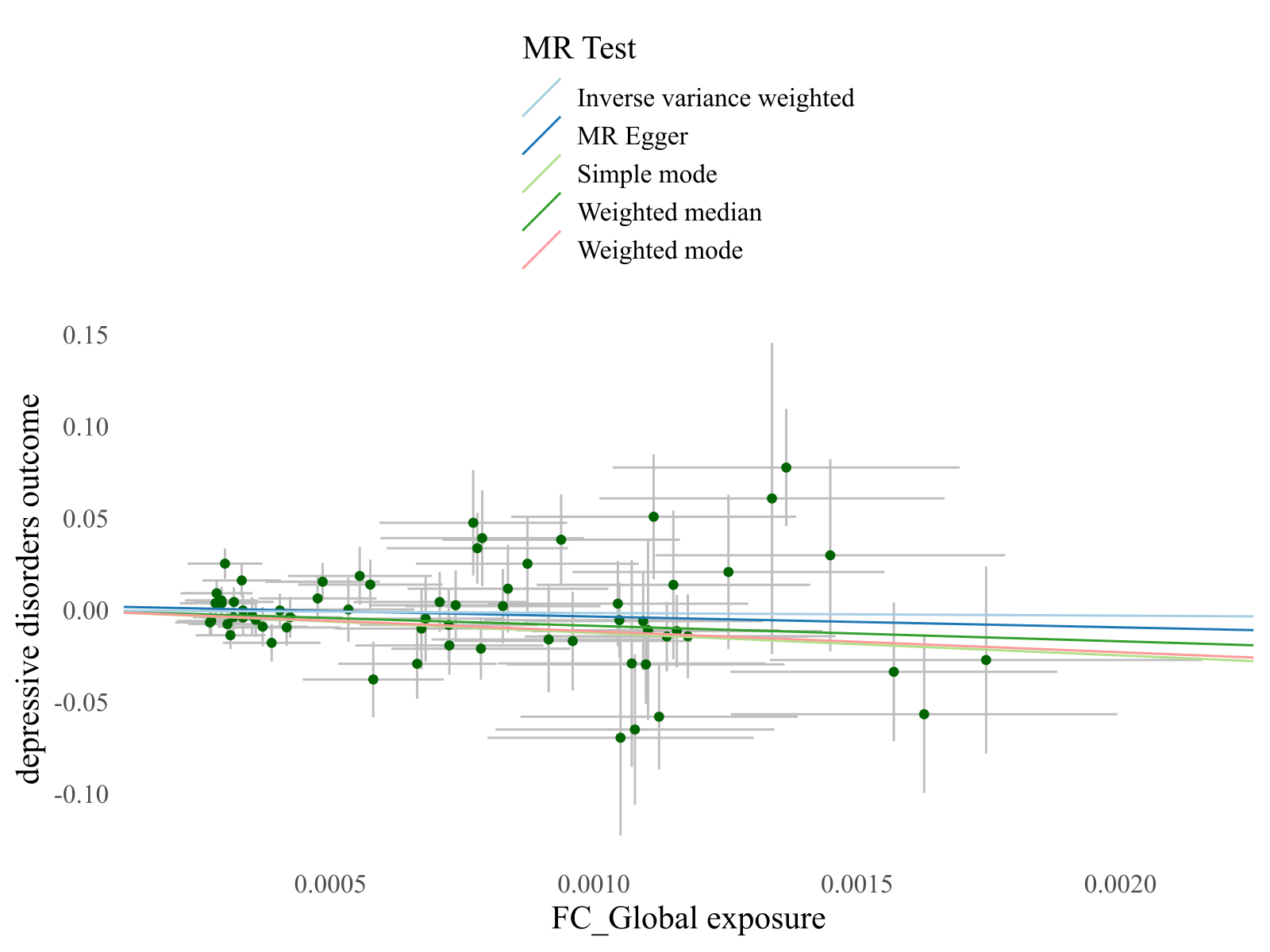


Fig.S6


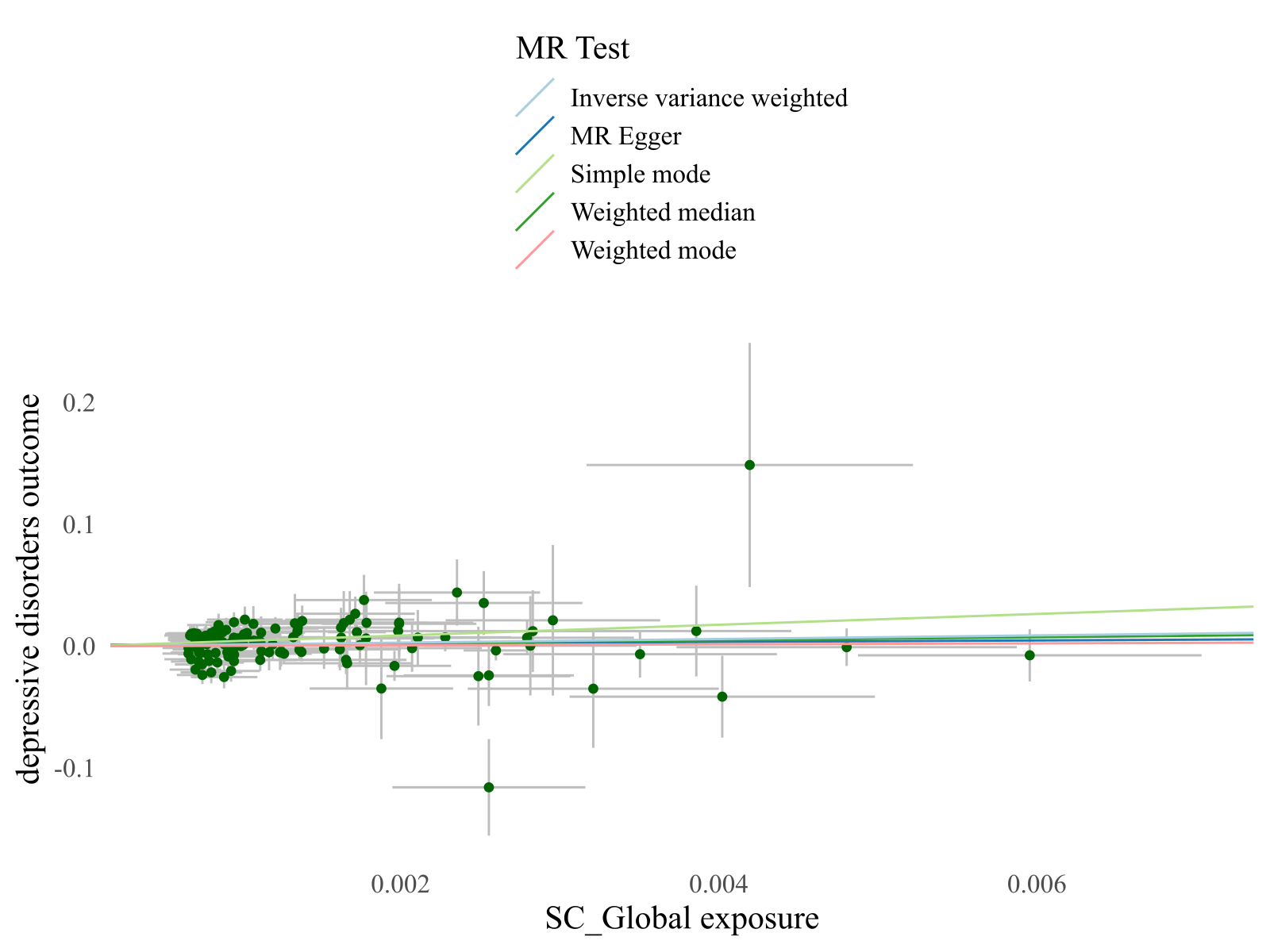


Fig.S7


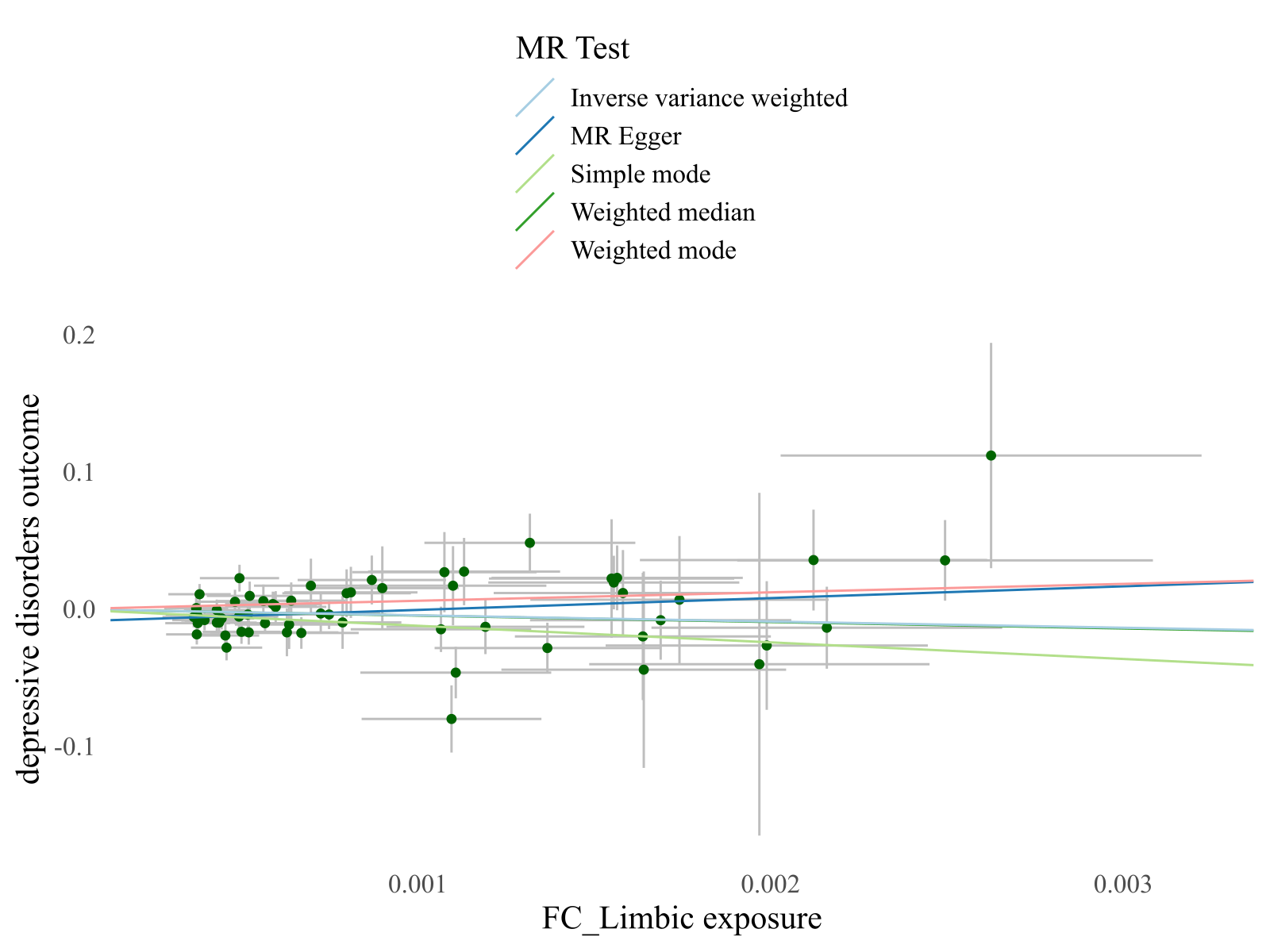


Fig.S8


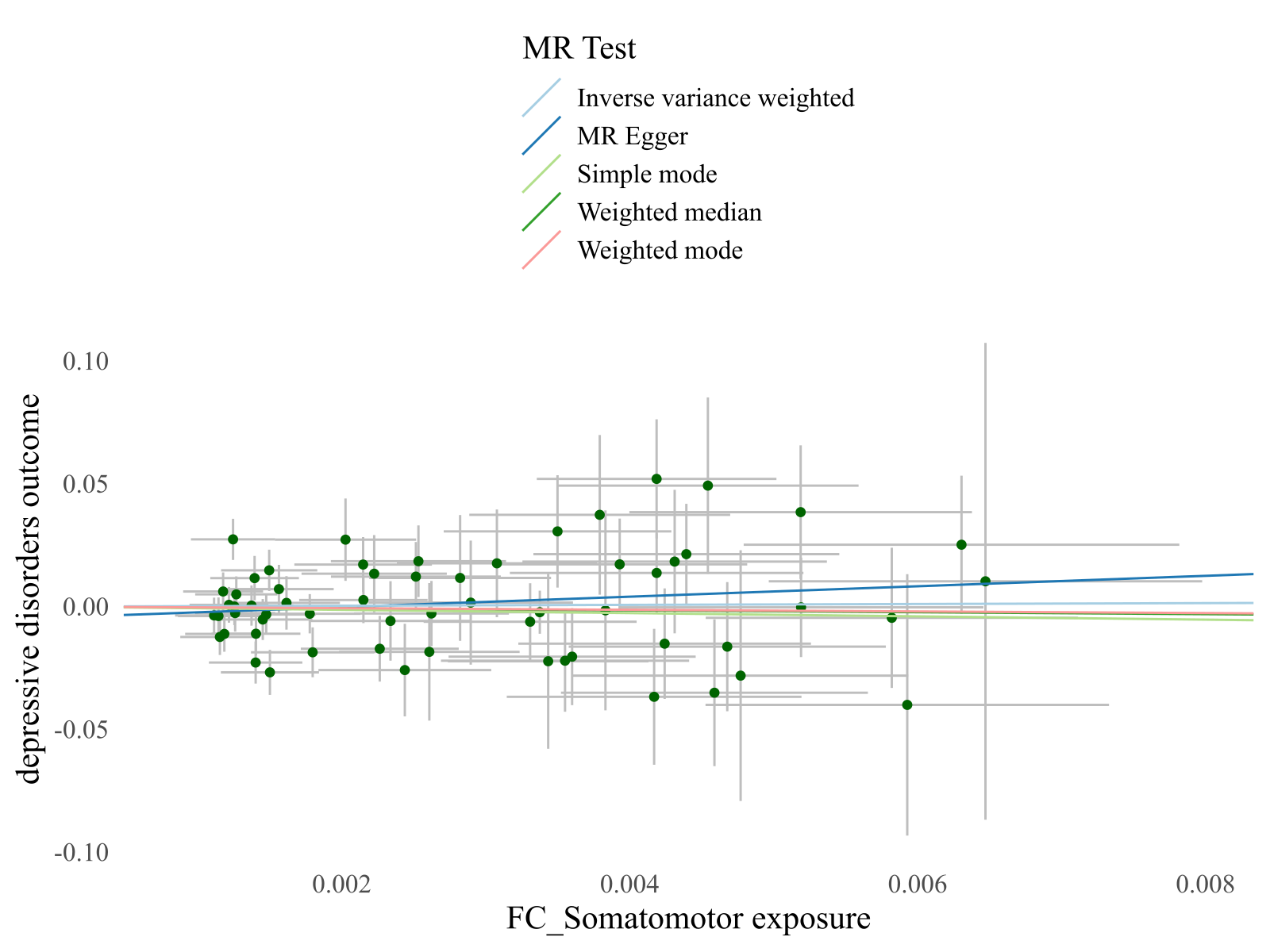


Fig.S9


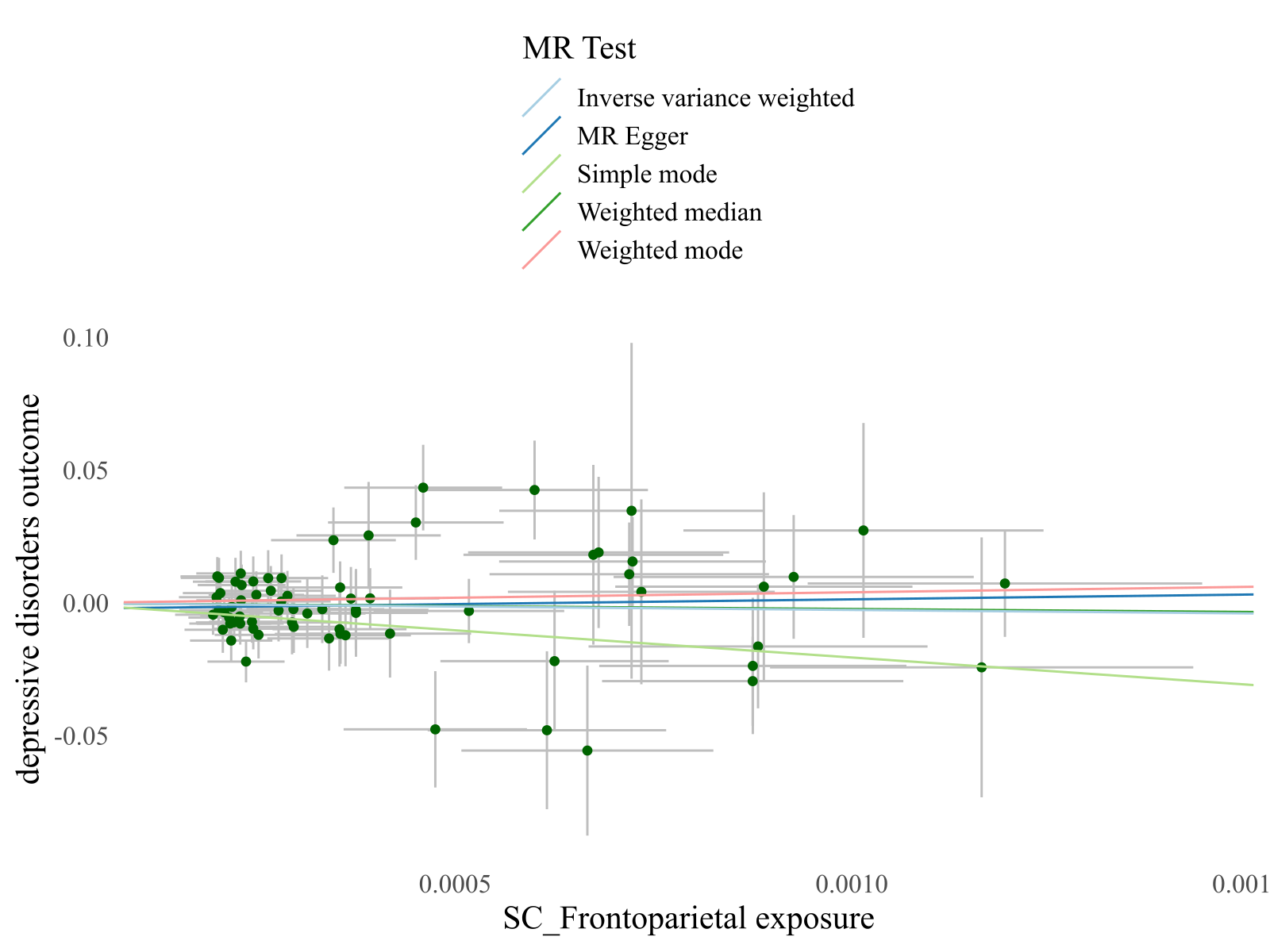


Fig.S10


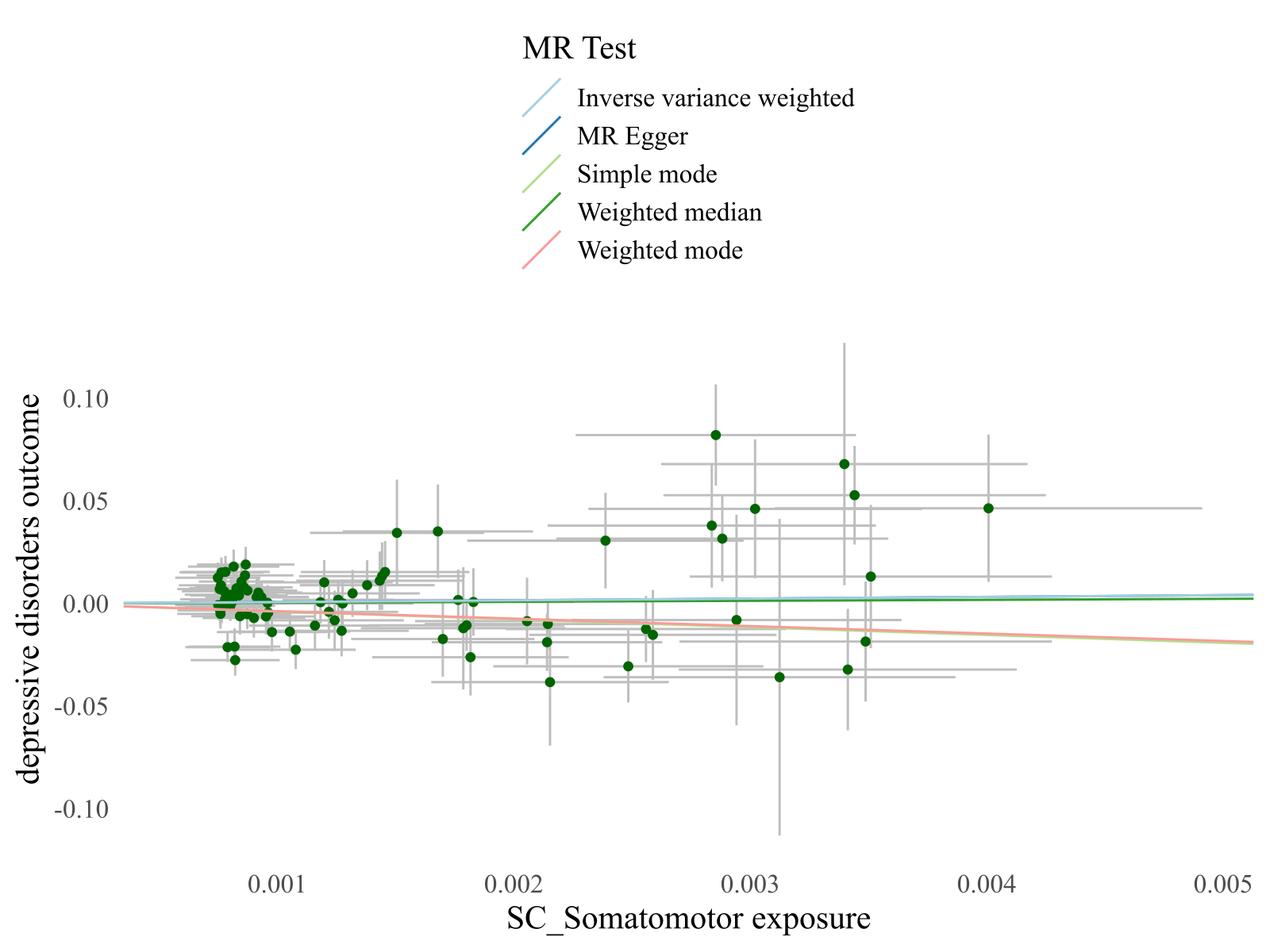


Fig.S11


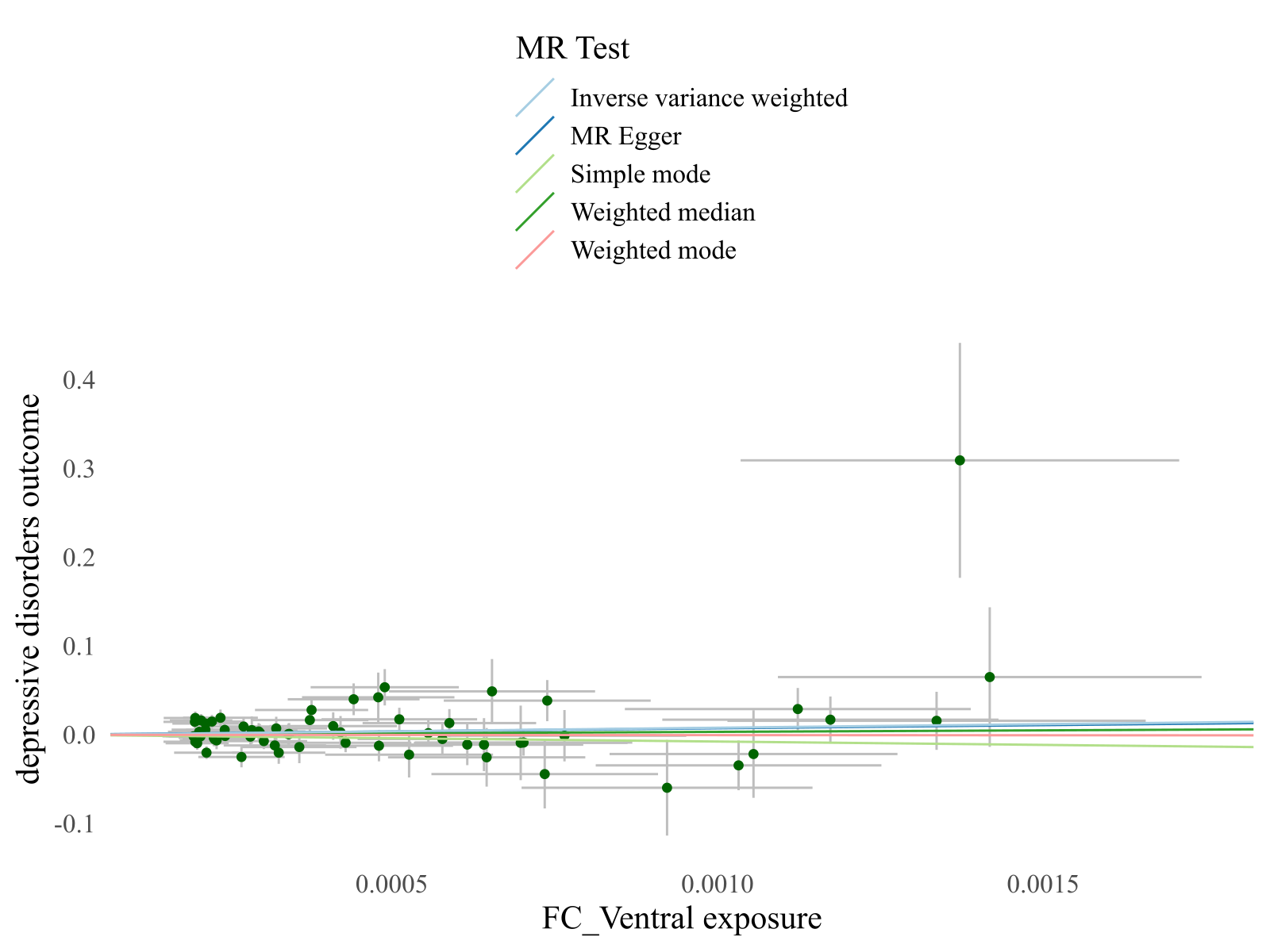


Fig.S12


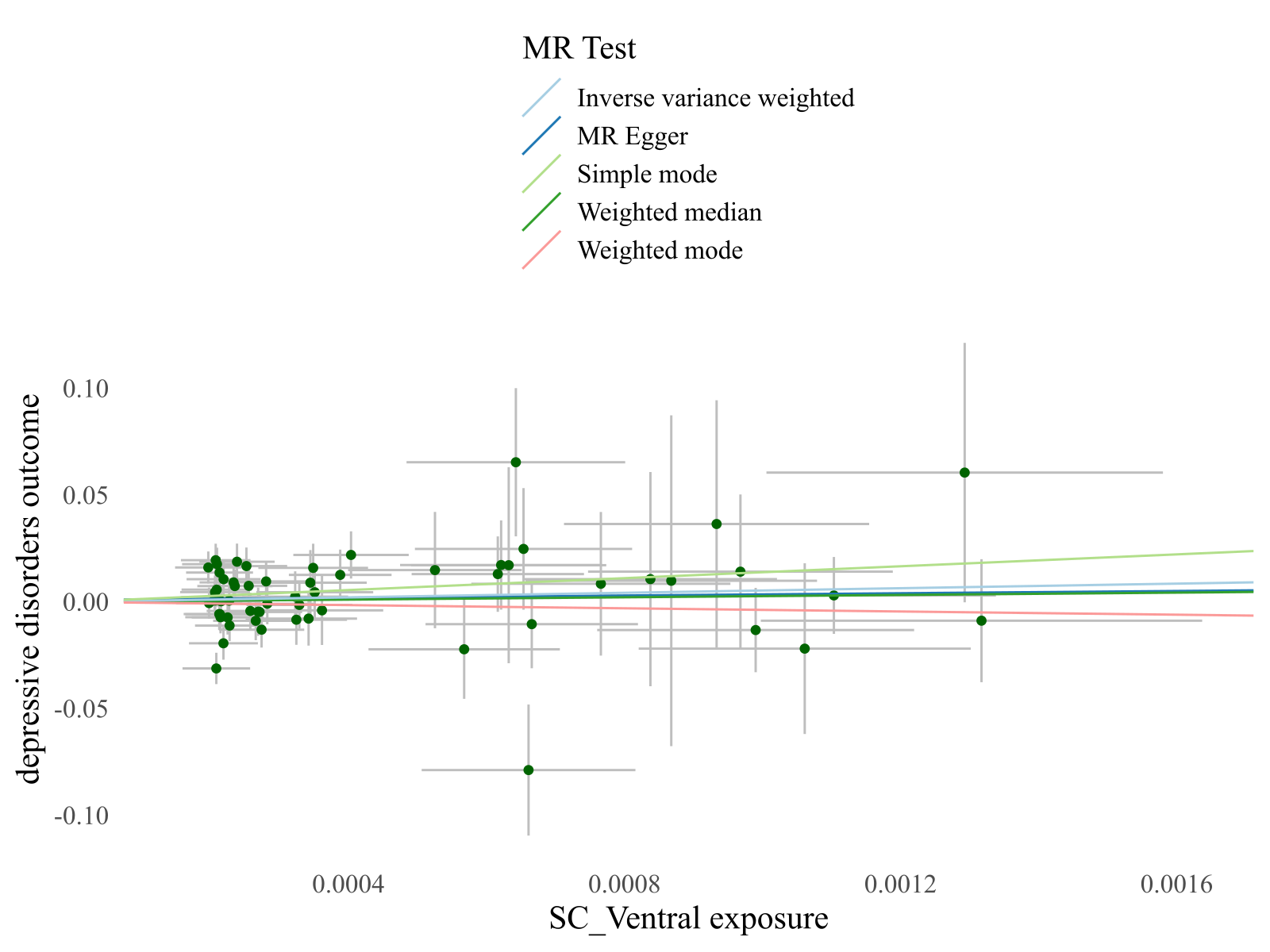


Fig.S13


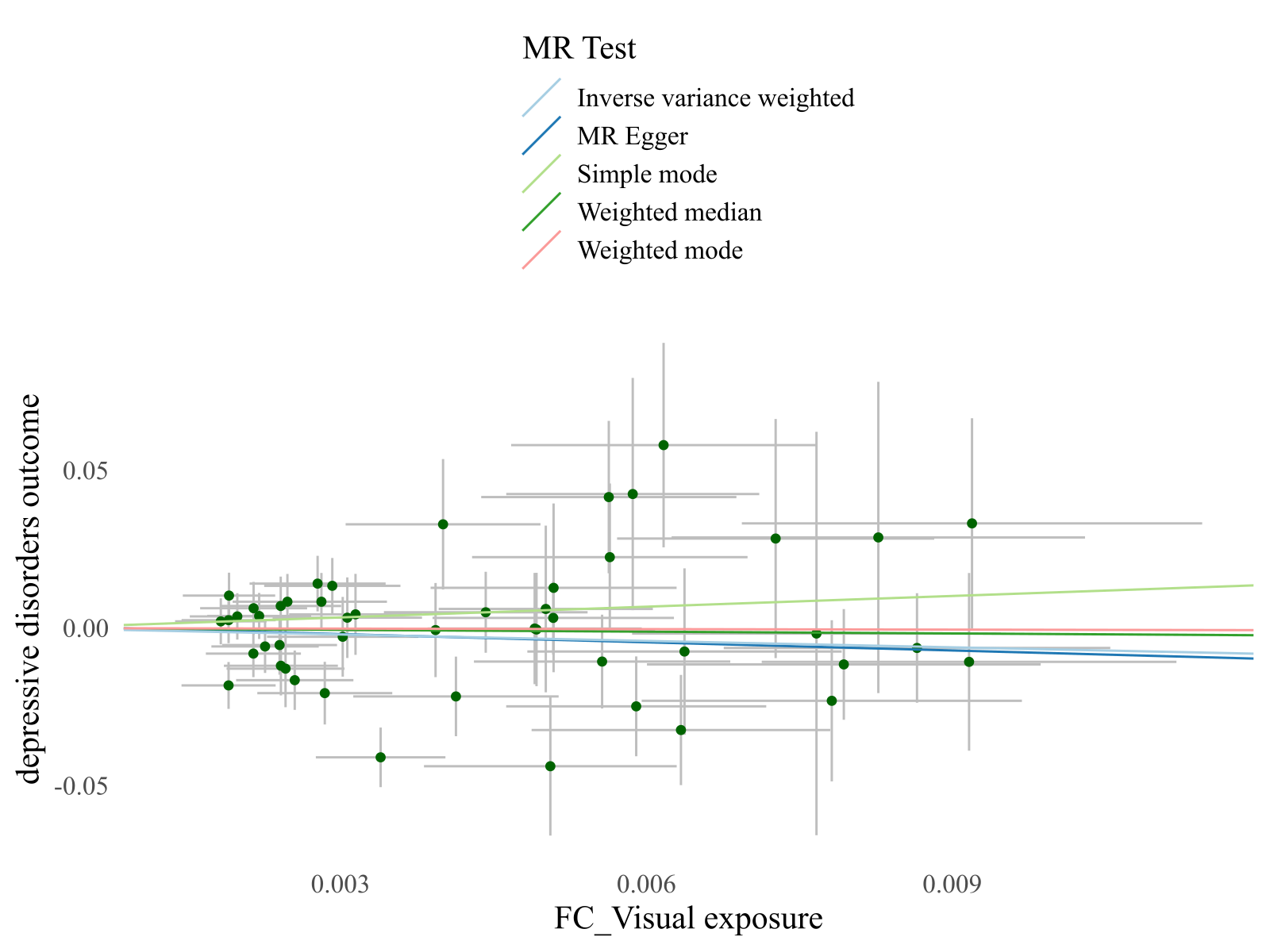


Fig.S14


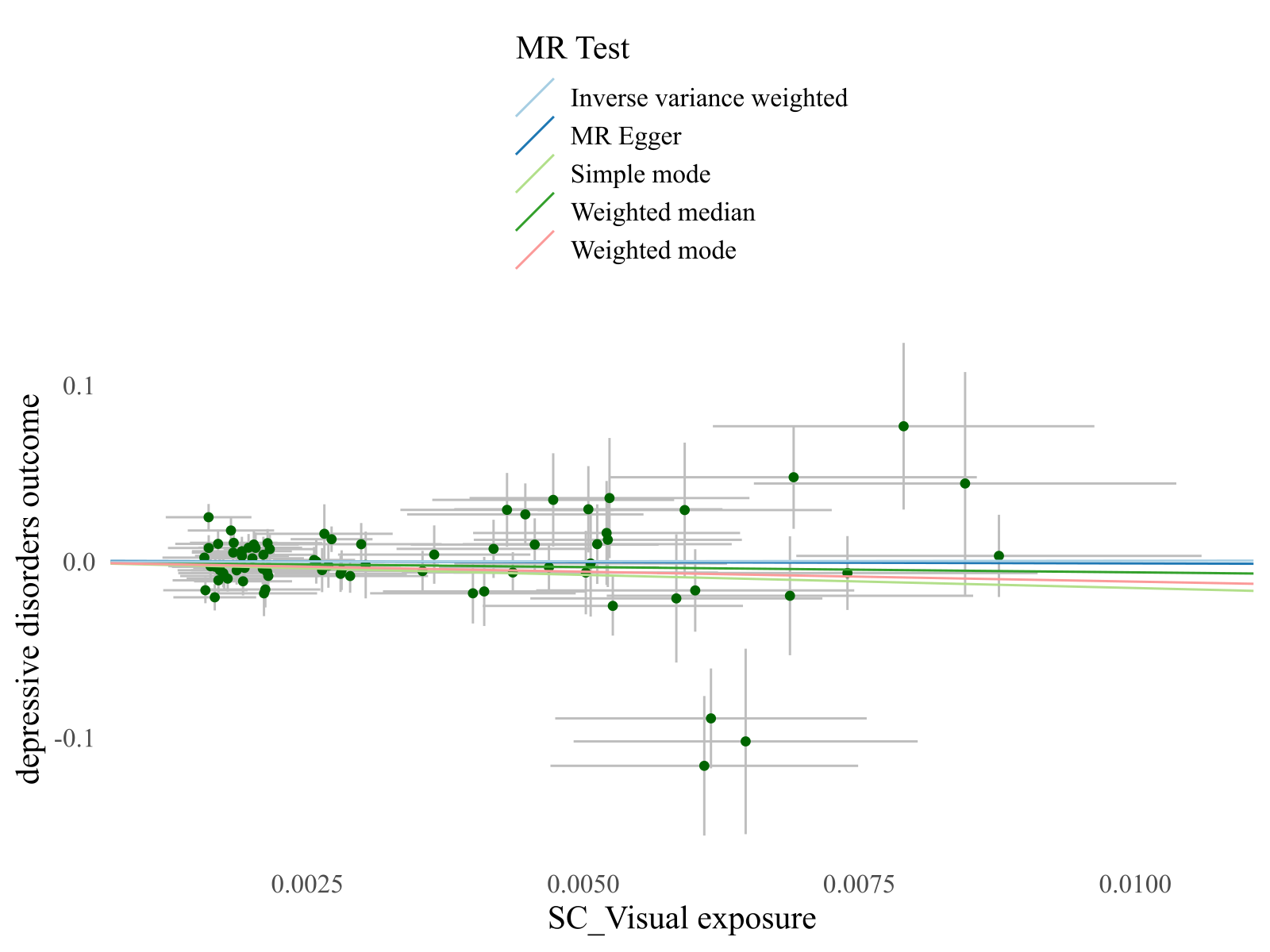


Fig.S15


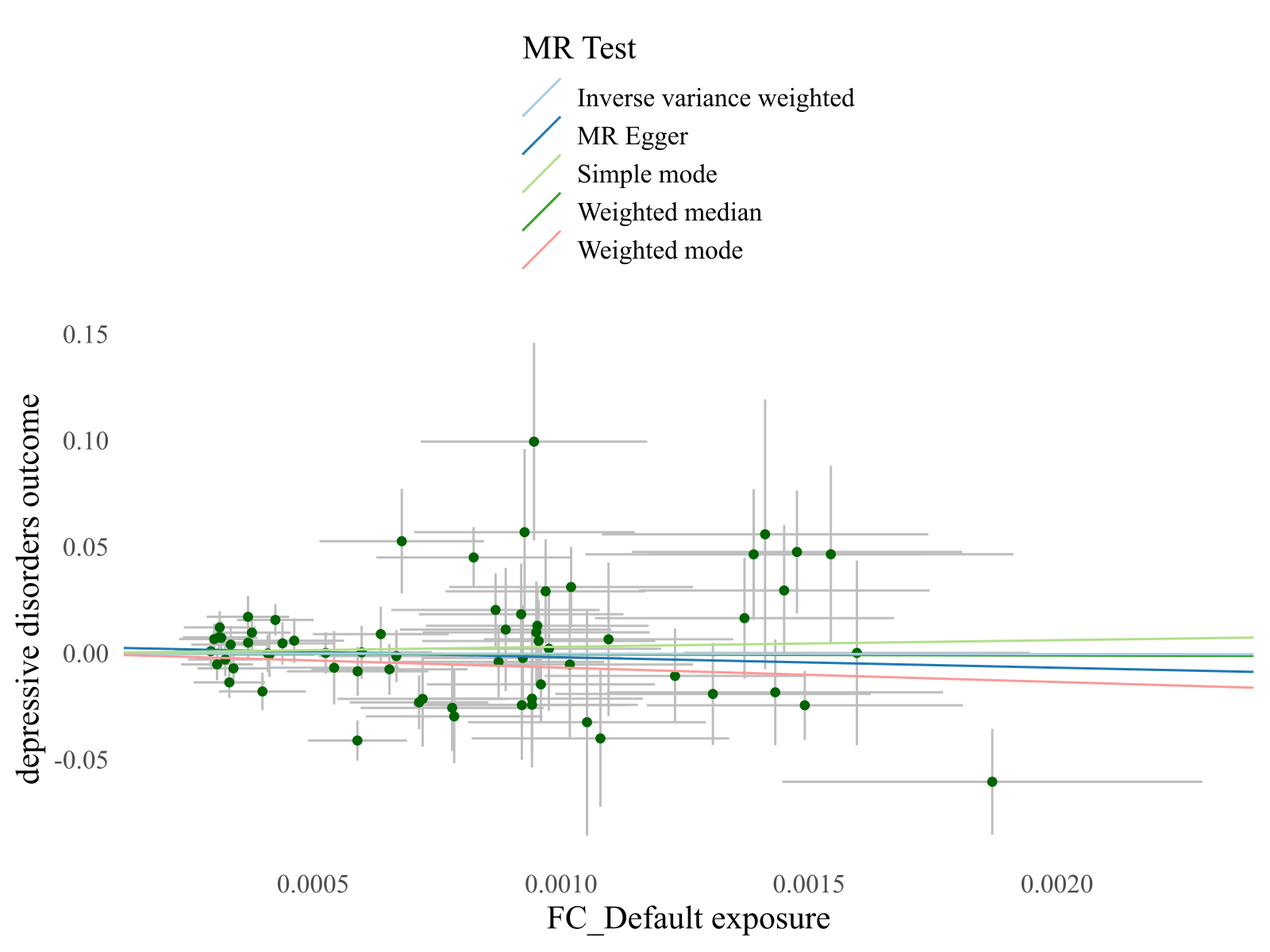


Fig.S16


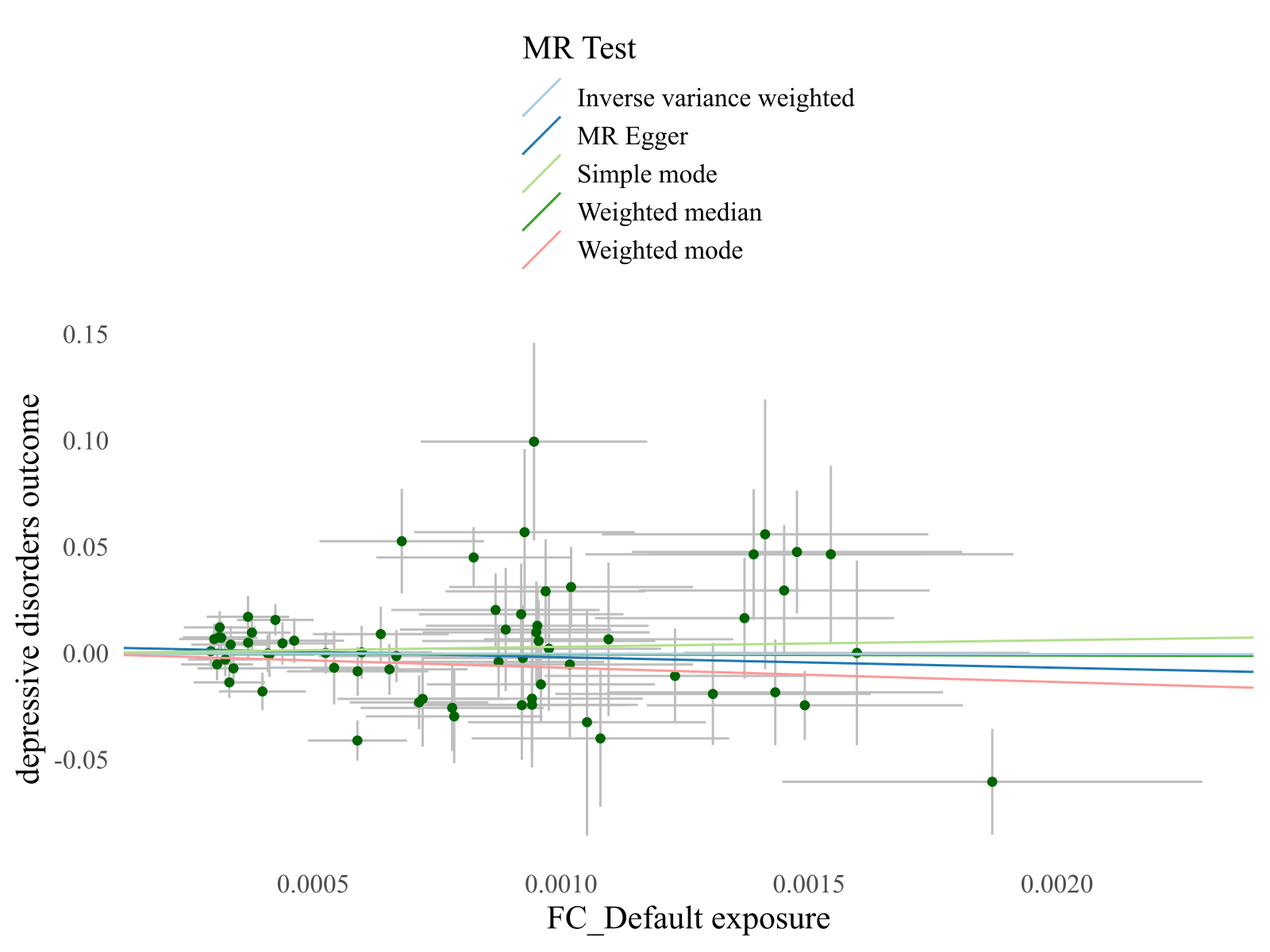


Fig.S17


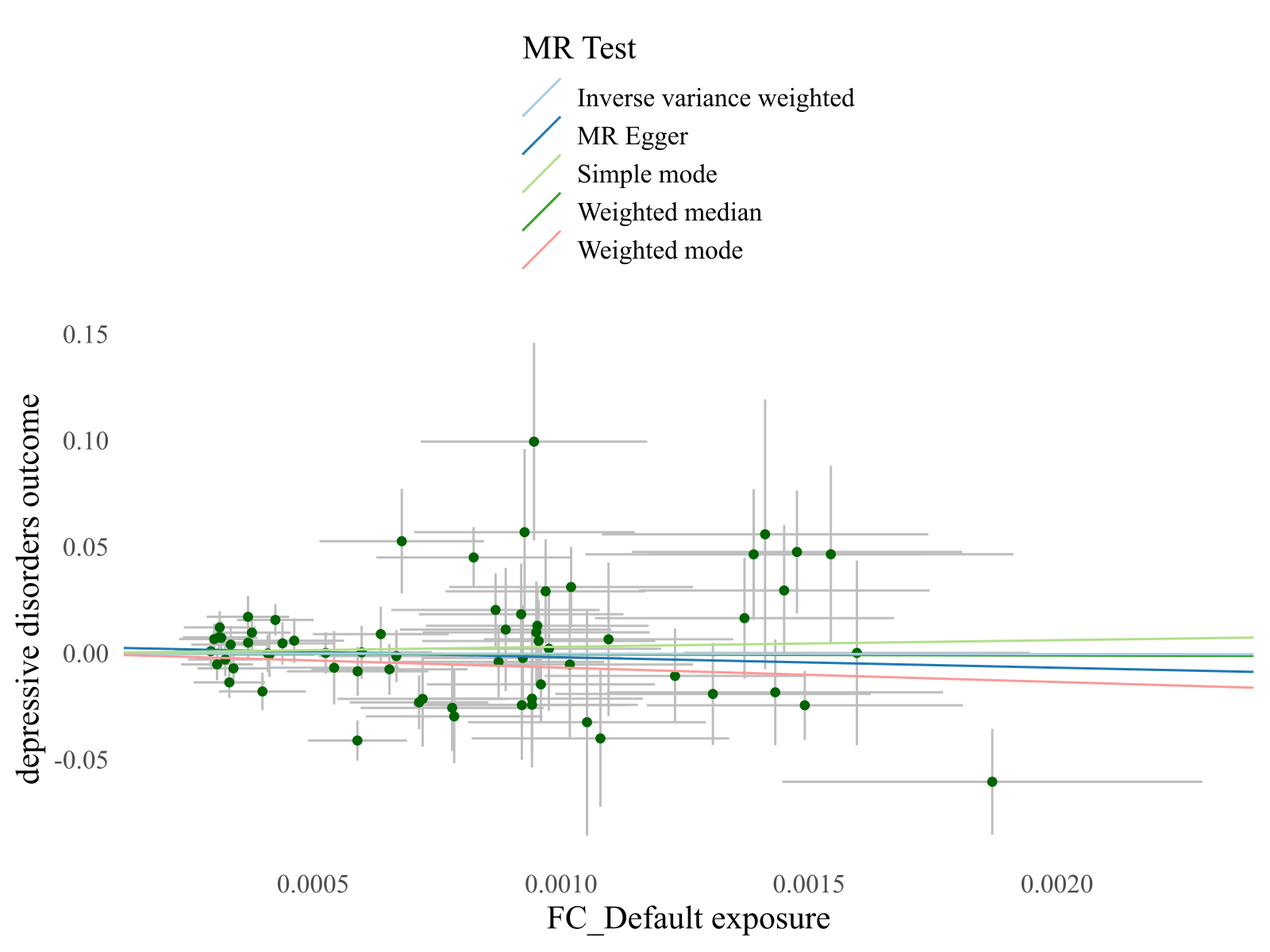


Fig.S18


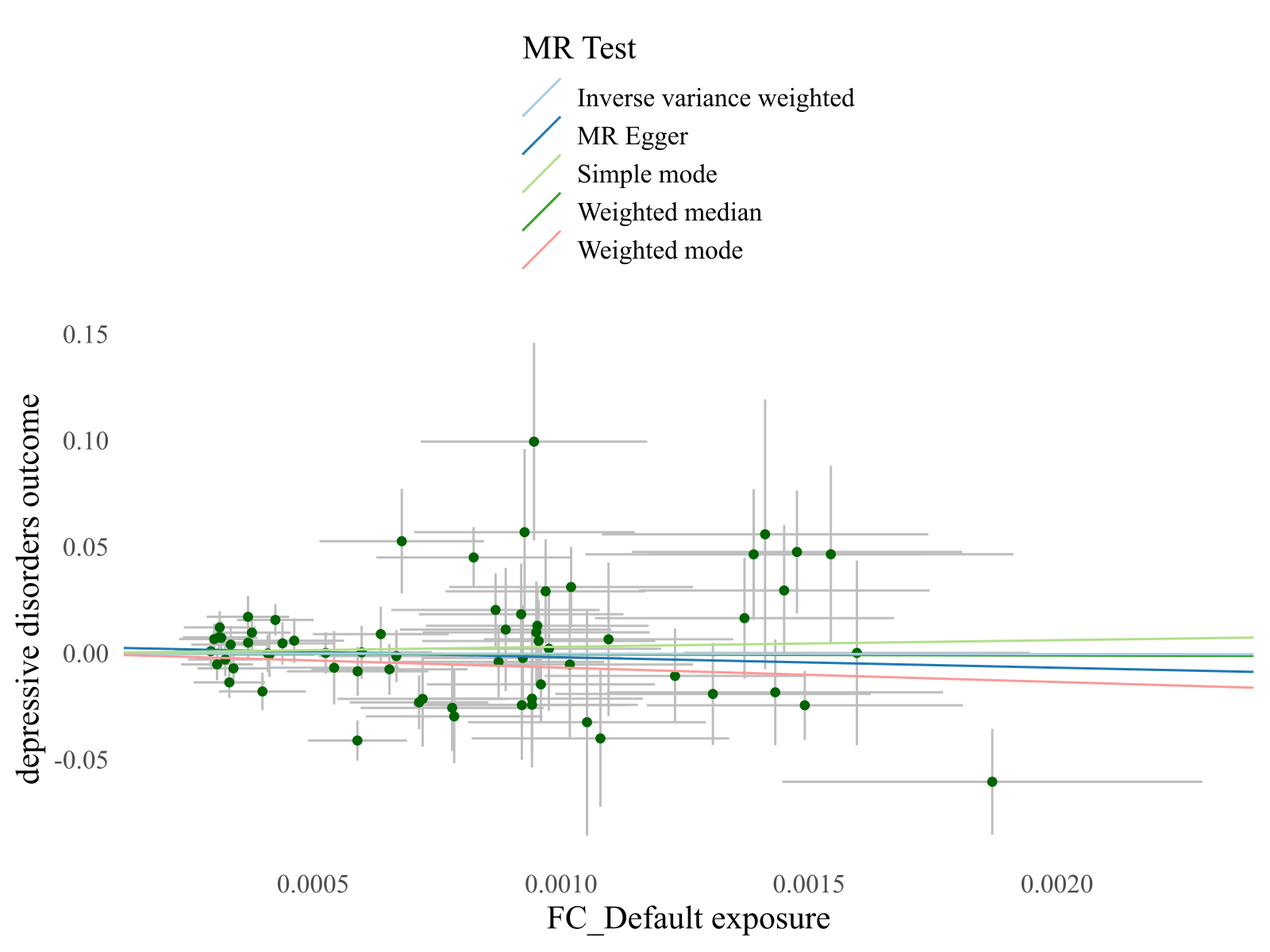


Fig.S19


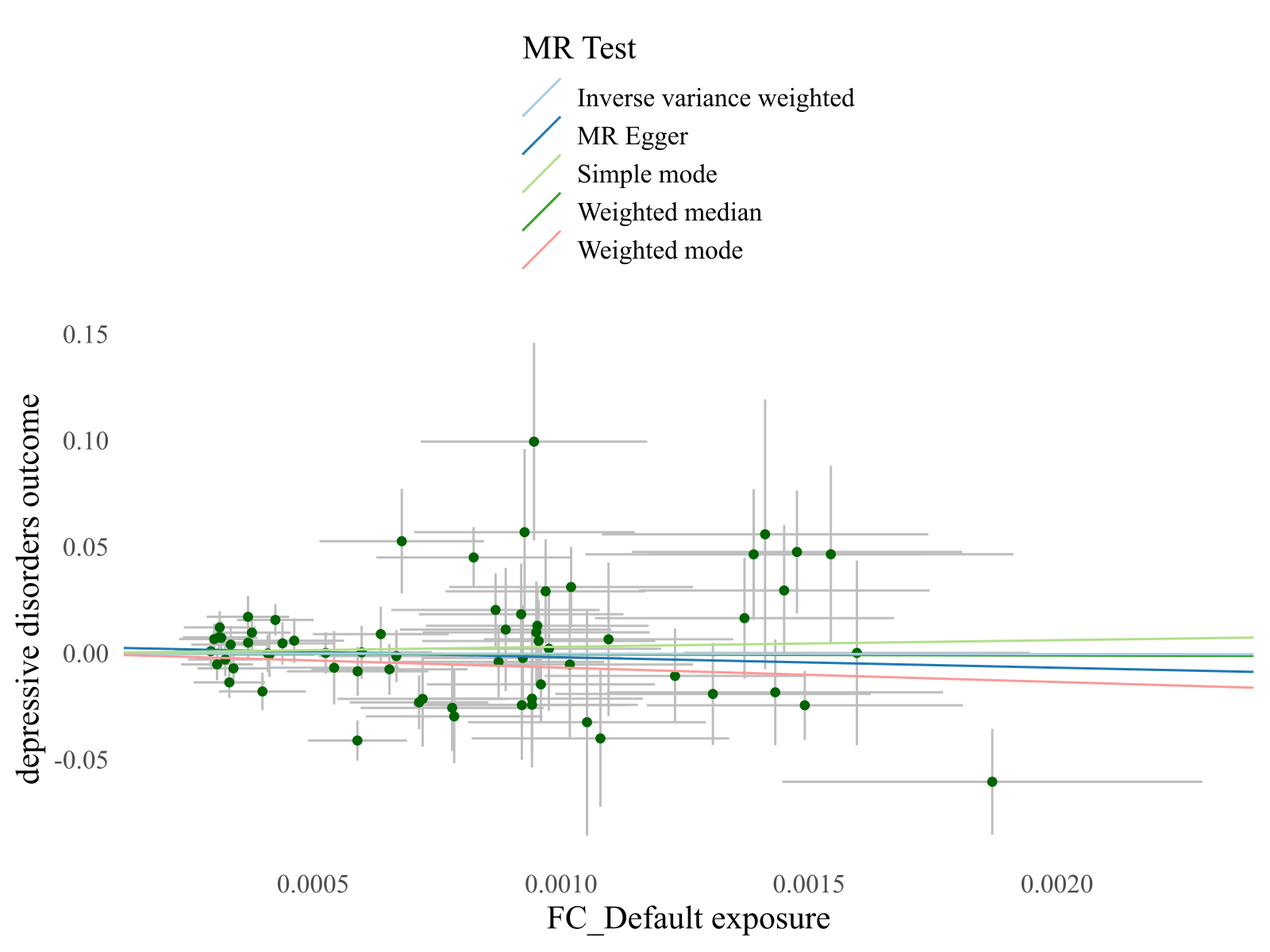


Fig.S20


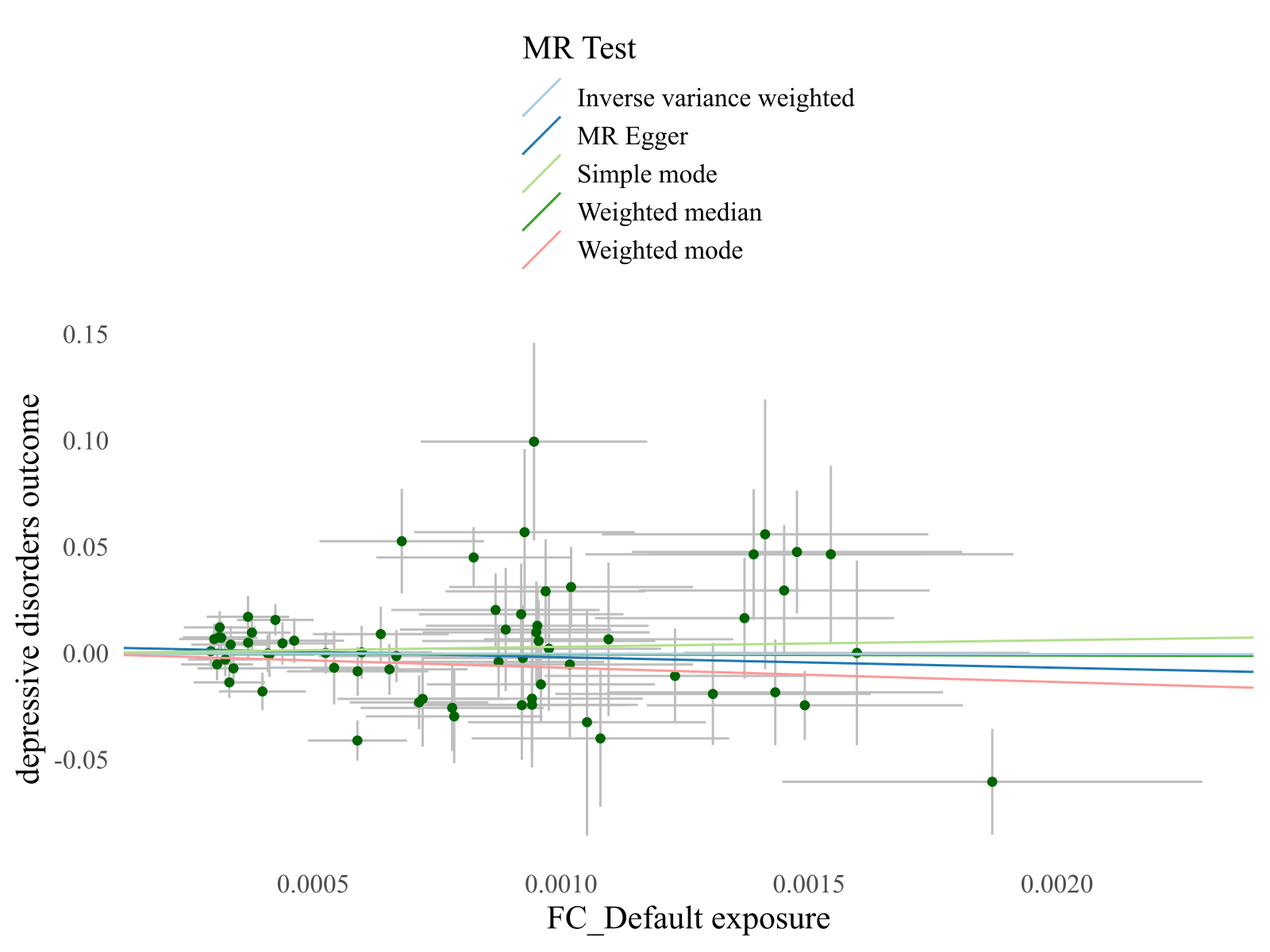


Fig.S21


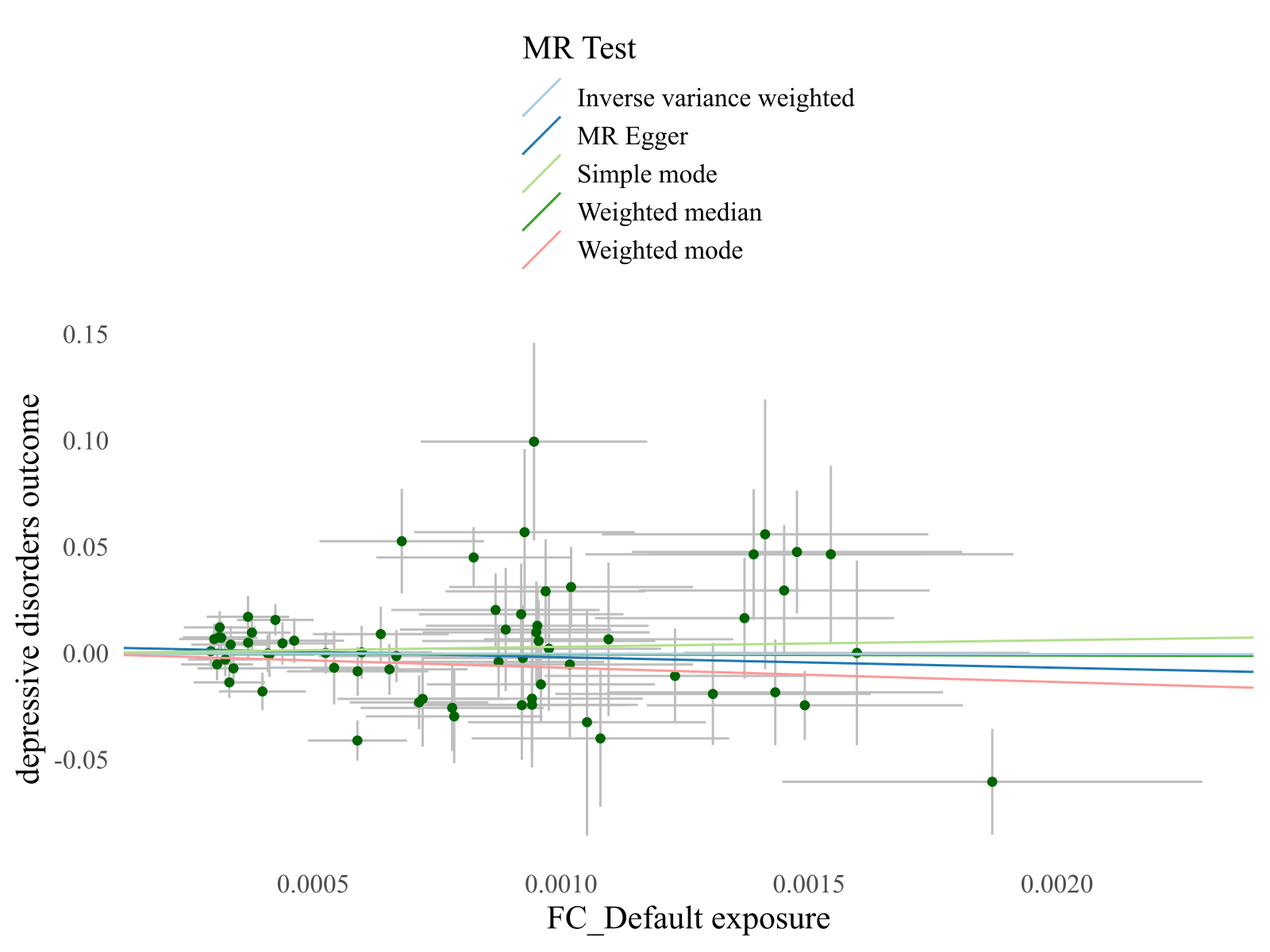


Fig.S22


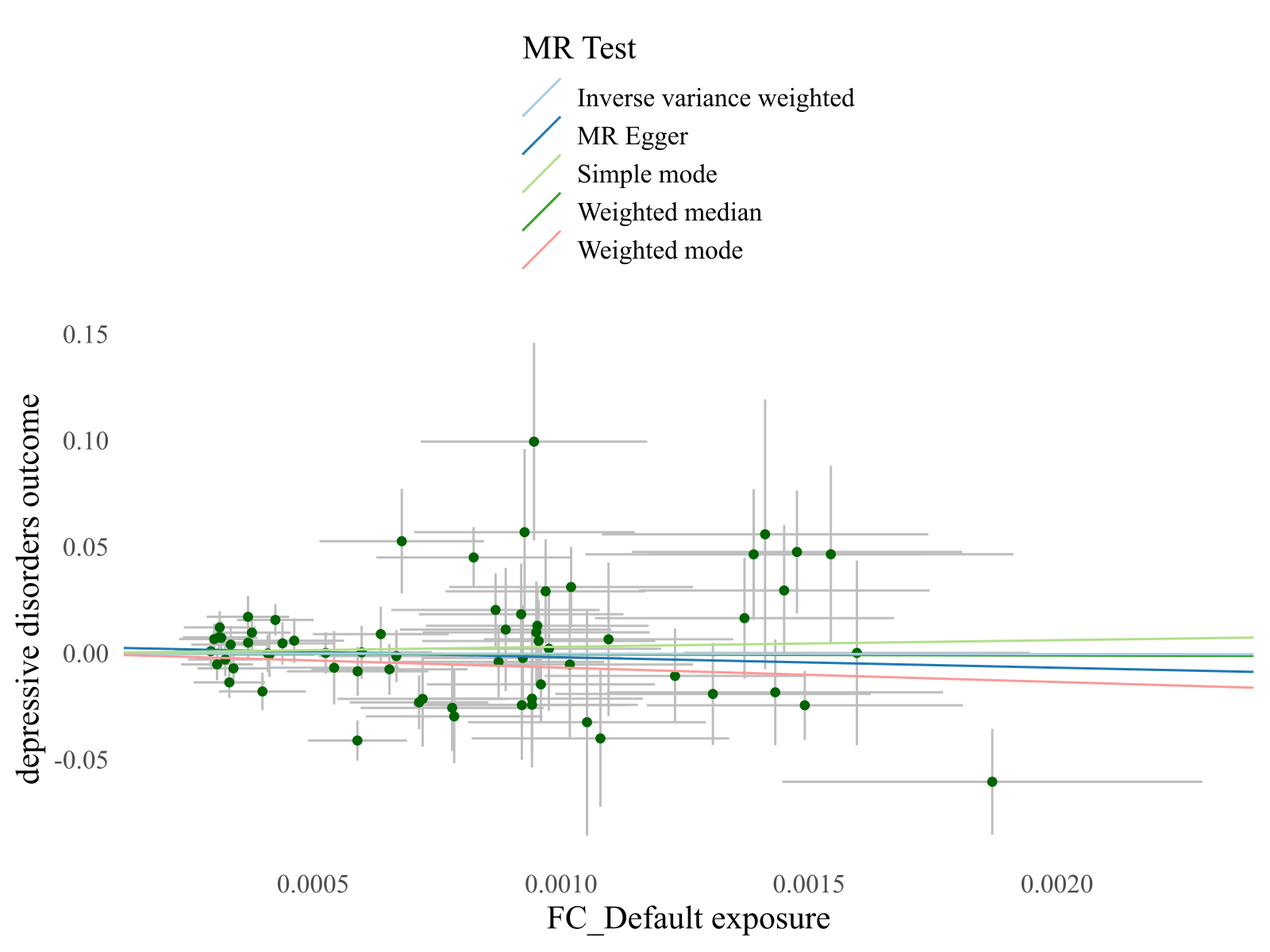


Fig.S23


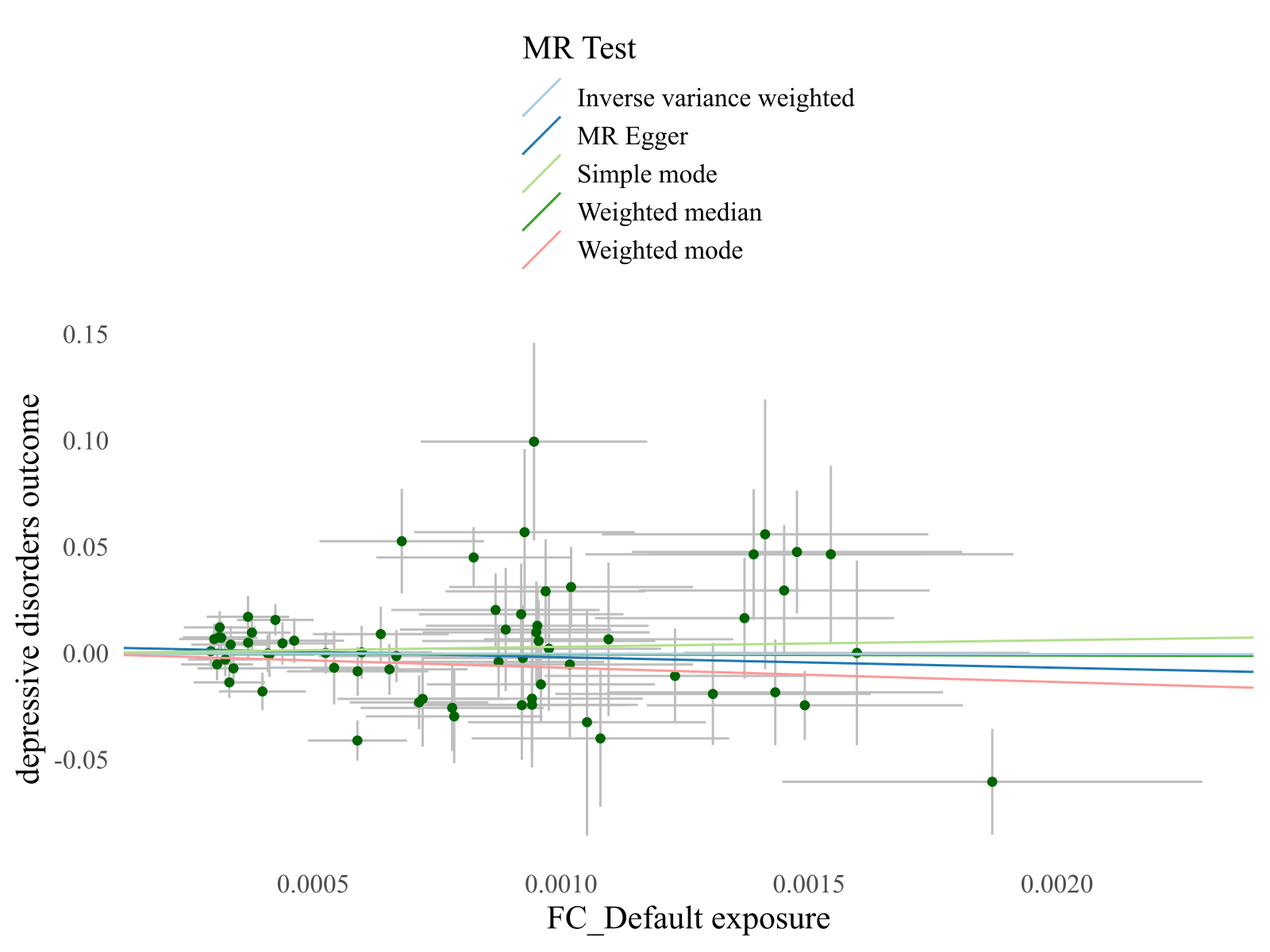


Fig.S24


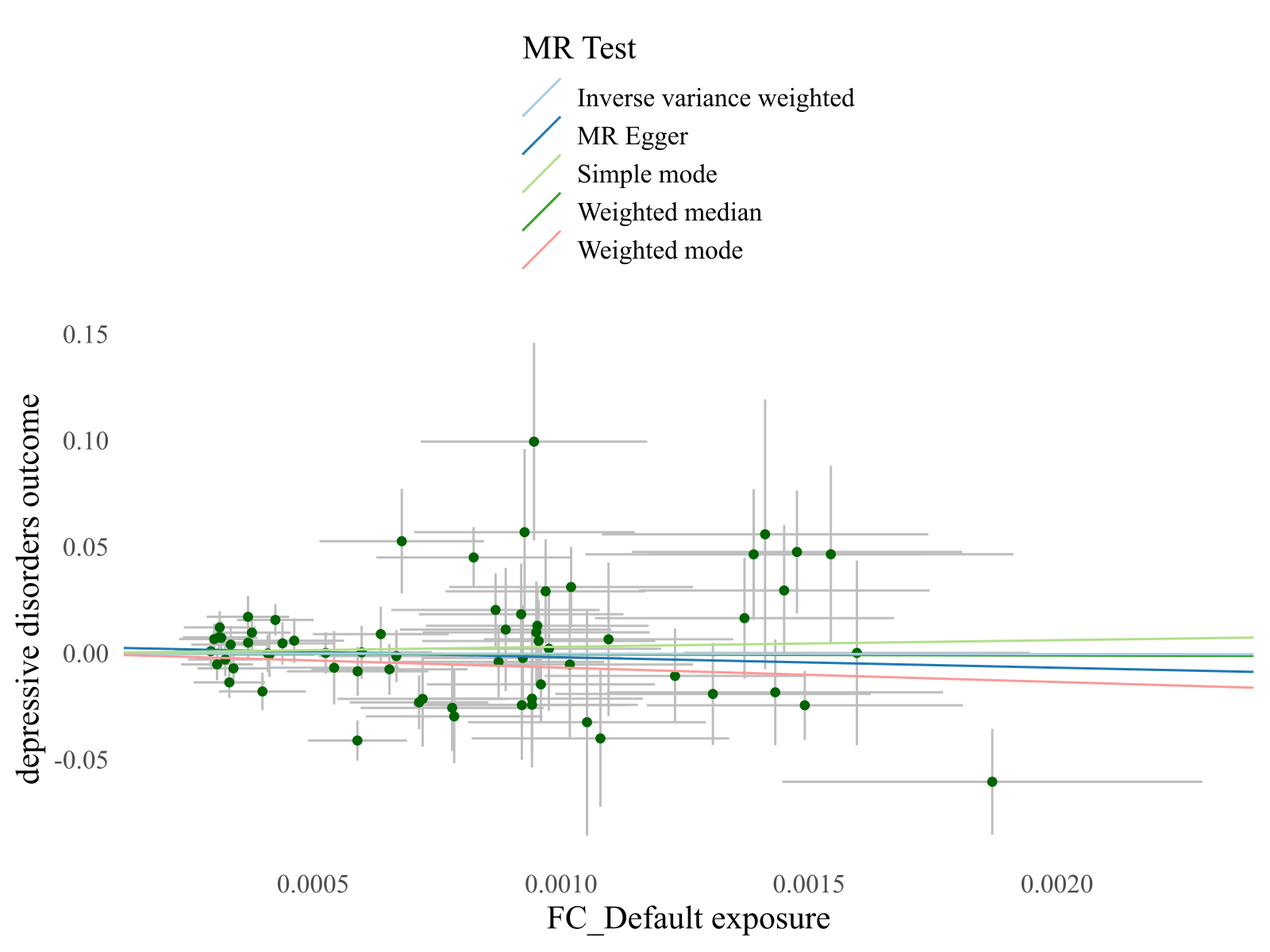


Fig.S25


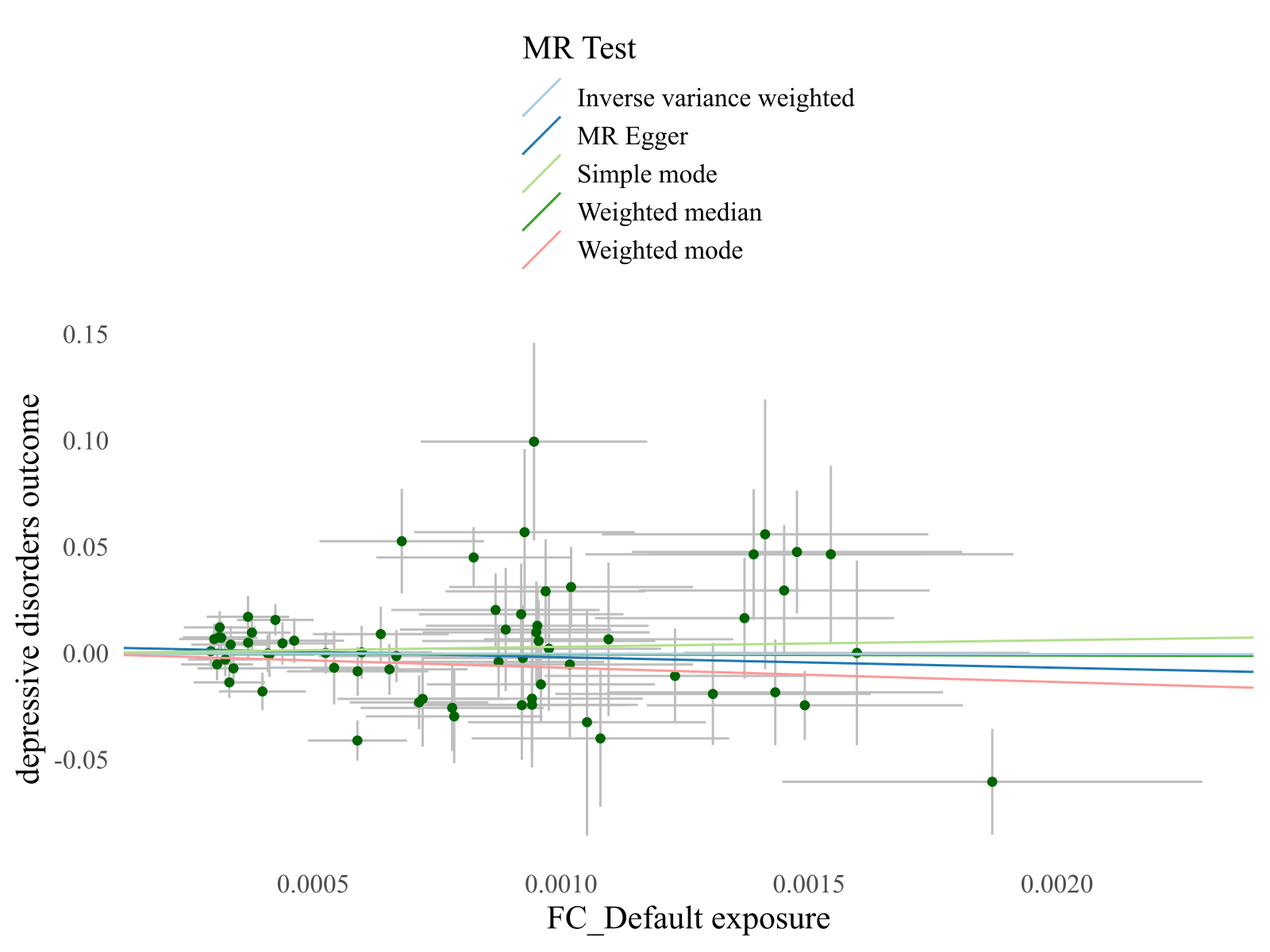


Fig.S26


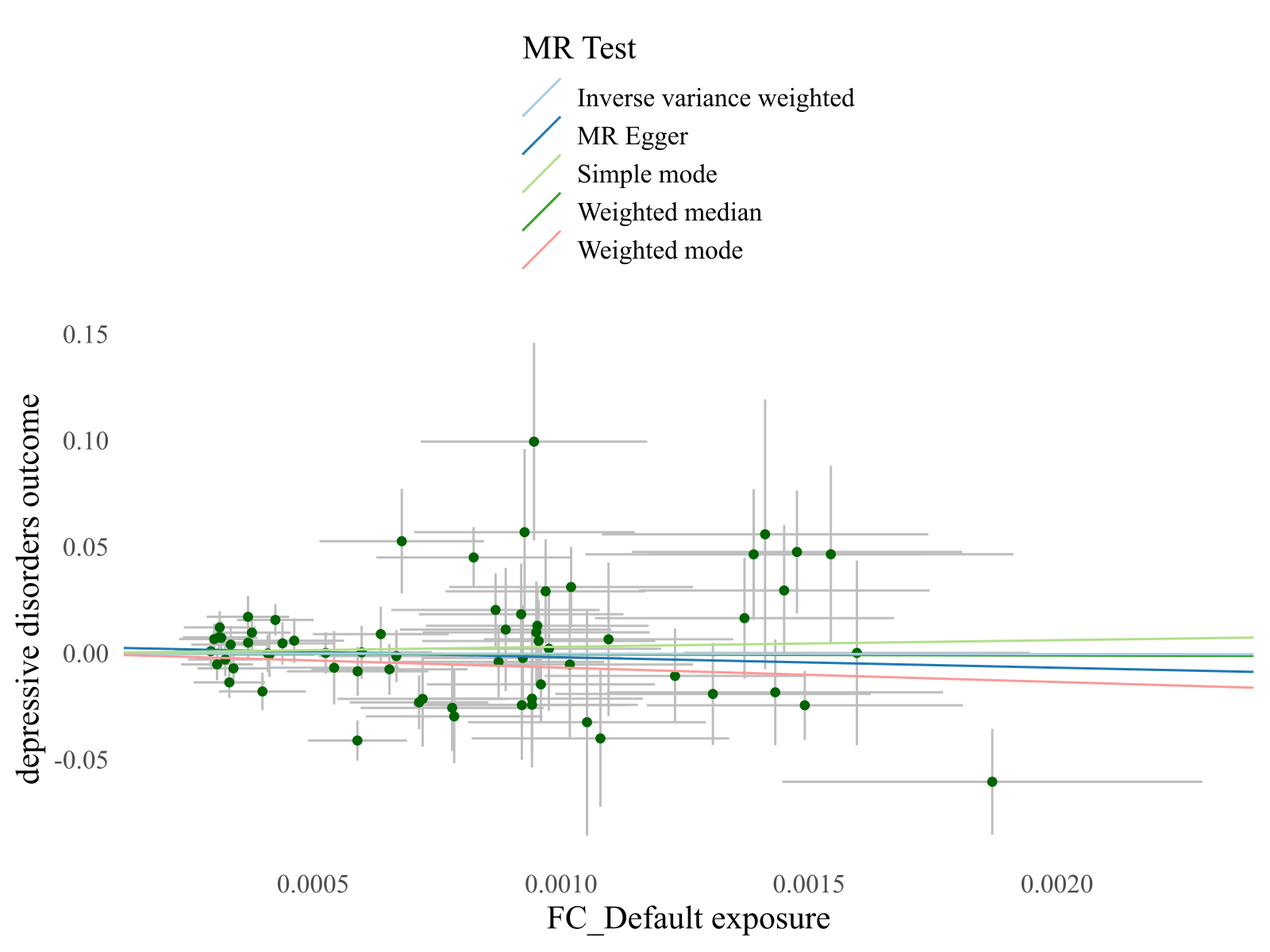


Fig.S27


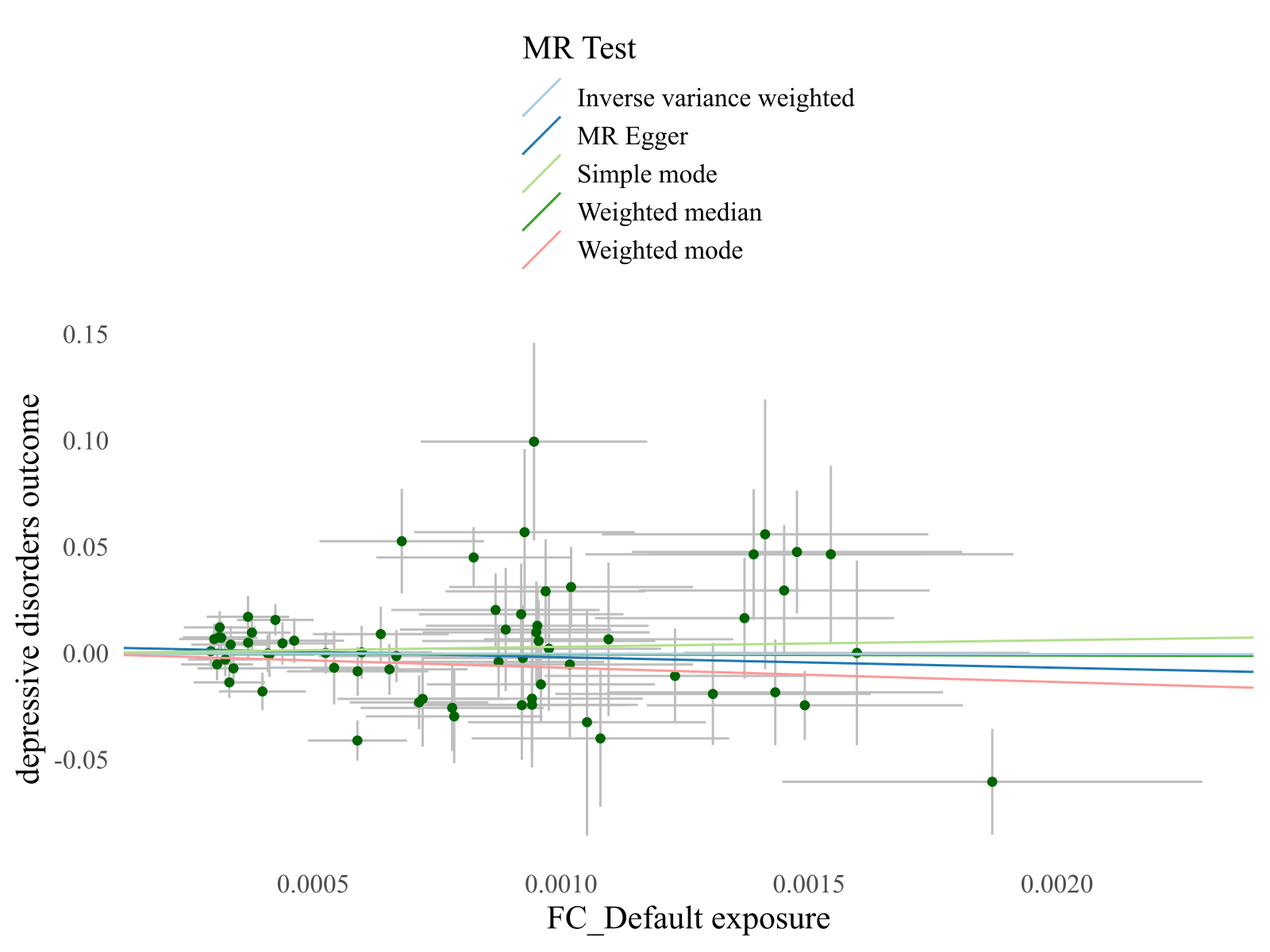


Fig.S28


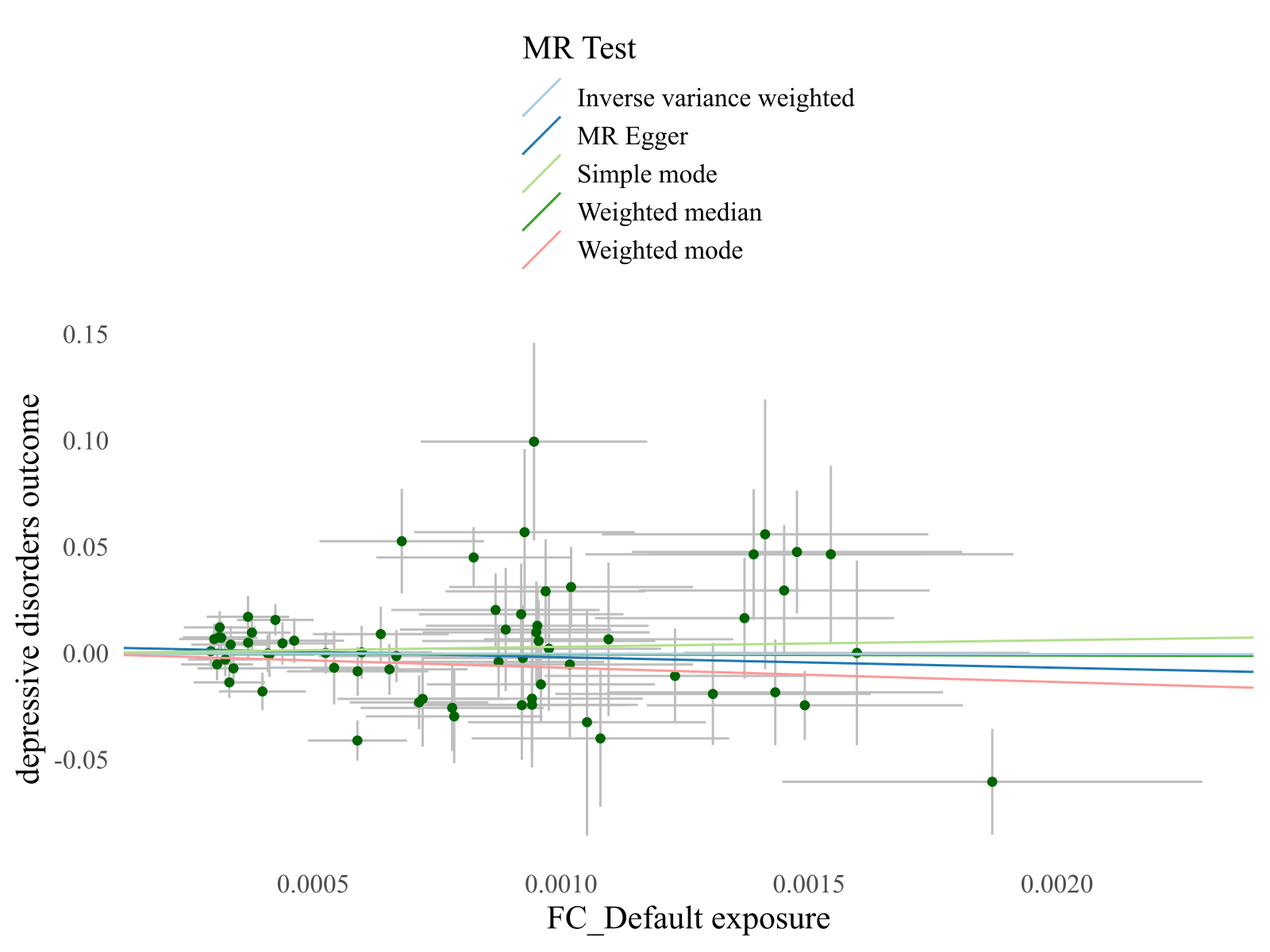


Fig.S29


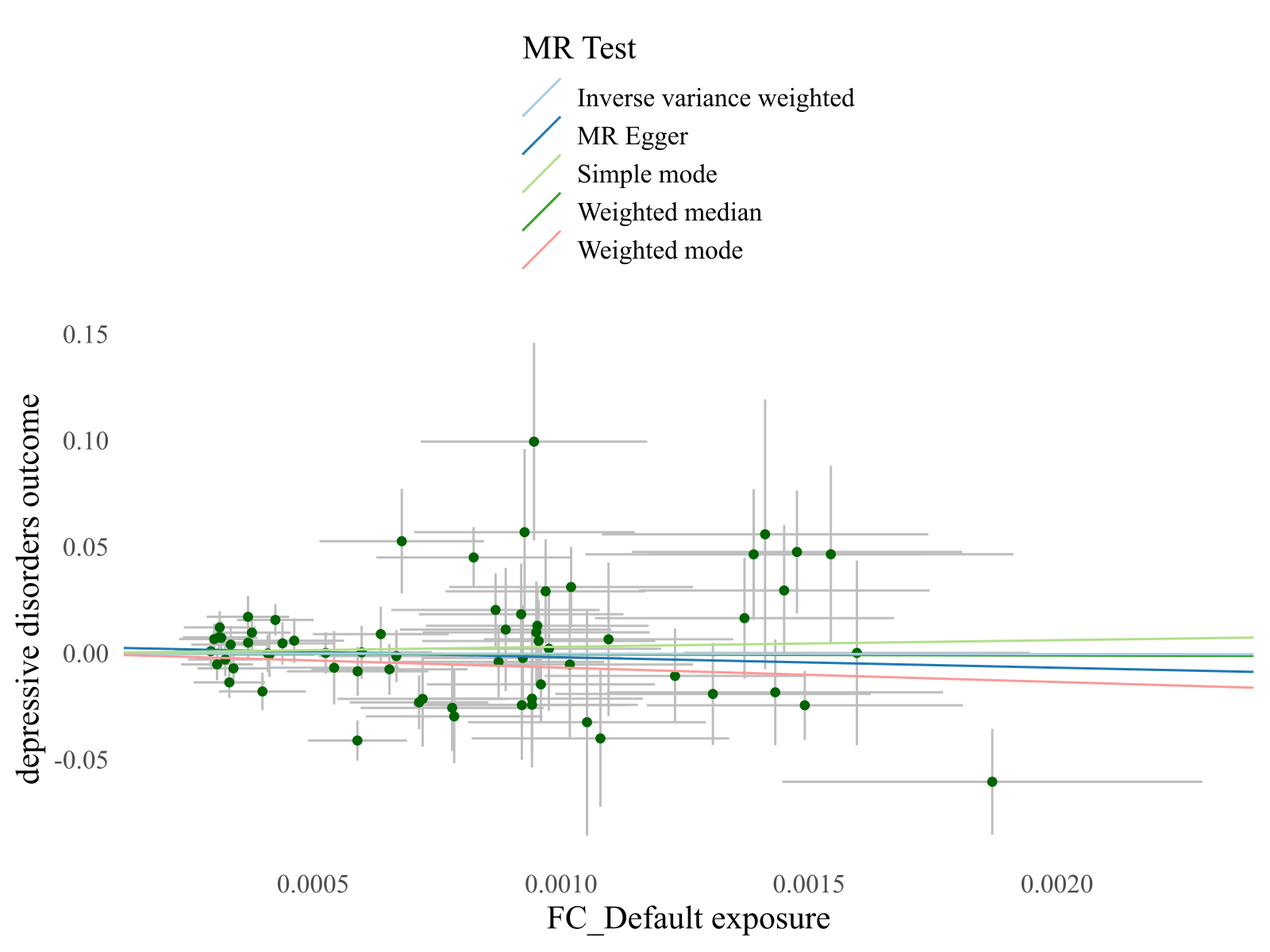


Fig.S30


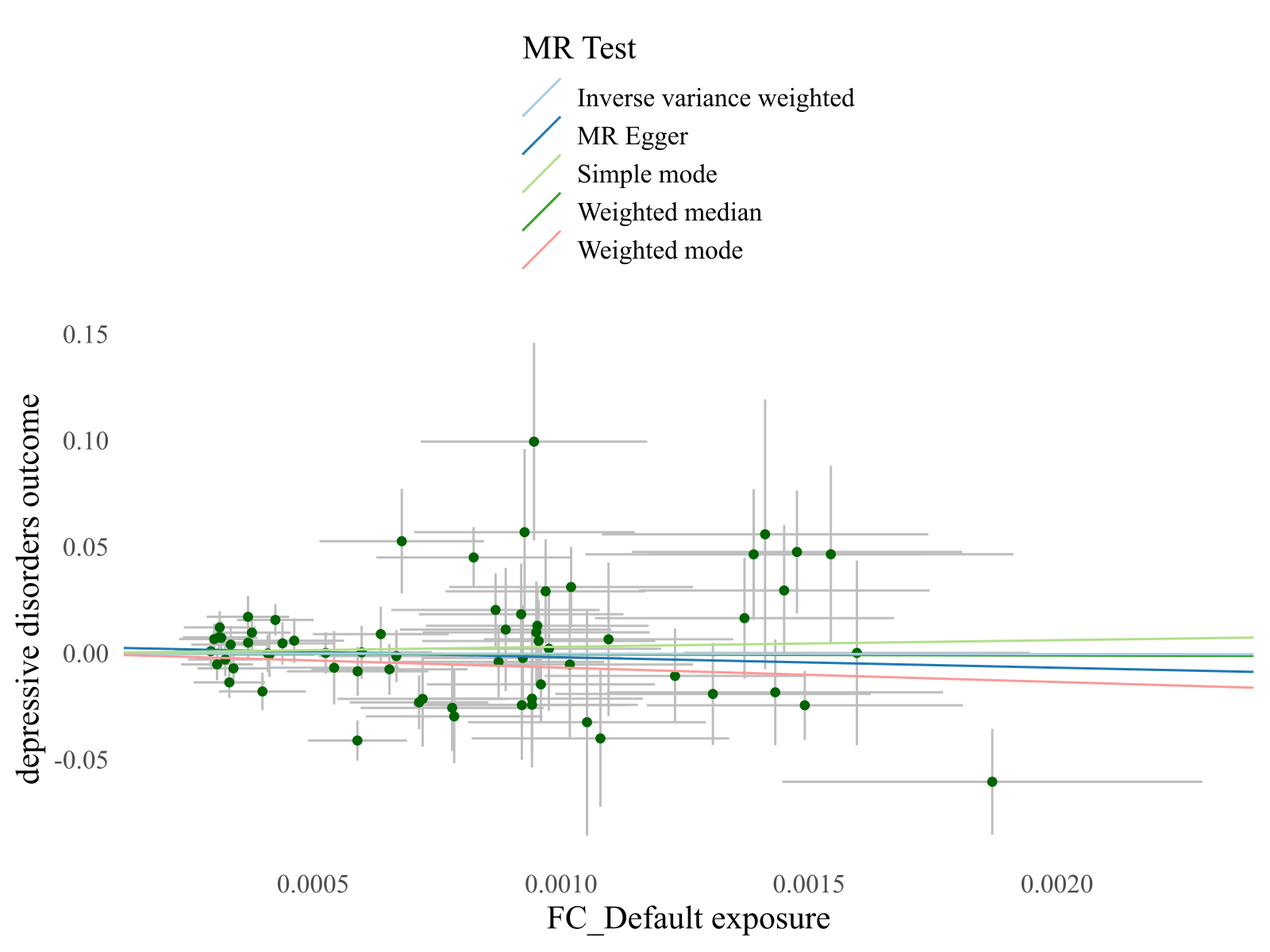

Supplement: Supplementary file 18 — Supplementary Material 18: The scatterplots of specific negative results [file 12888_2024_5857_MOESM18_ESM.docx]
